# The tango of immune and neural cells: orchestrating neuroinflammation mediated brain disorders

**DOI:** 10.3389/fimmu.2026.1827437

**Published:** 2026-05-21

**Authors:** Yimao Wu, Weiqi Lyu, Xiaofei Xie, Xiao Fu, Liao Zhang, Junfu Pan

**Affiliations:** 1 Zhejiang Provincial Clinical Research Center for Mental Disorder, The Affiliated Kangning Hospital of Wenzhou Medical University, Wenzhou, China; 2 Second Clinical Medical College, Guangdong Medical University, Dongguan, China; 3 First Clinical Medical College, Guangdong Medical University, Zhanjiang, China; 4 School of Mental Health, Wenzhou Medical University, Wenzhou, China

**Keywords:** Alzheimer’s disease, clinical translation, immune cell-neural cell interaction, molecular mediators, neuroinflammation, Parkinson’s disease

## Abstract

The bidirectional interaction between immune cells and neural cells is the core effector unit of neuroinflammation, determining whether the central nervous system (CNS) maintains homeostasis or develops disease. Under physiological conditions, this interaction supports CNS homeostasis through microglial surveillance, astrocytic metabolic support (including the astrocyte-neuron lactate shuttle and glutamate reuptake), and blood-brain barrier (BBB) integrity. Under pathological states, dysregulated immune-neural crosstalk drives neuroinflammation. Damaged neurons activate microglia and astrocytes, which in turn secrete proinflammatory factors that impair neurons, reduce neurotrophic support, and disrupt BBB integrity. This interaction operates through cell surface receptor-ligand systems and soluble signals. Representative diseases including Alzheimer’s disease (AD), Parkinson’s disease (PD), depression, and schizophrenia share common inflammatory features such as glial cell activation, BBB damage, and synaptic dysfunction, while exhibiting disease-specific pathological mechanisms. Clinical translation progress has been made in developing biomarkers for diagnosis, targeting immune cells and neural cells for therapy, and exploring emerging interventions like immunometabolic regulation and cell therapy. However, gaps remain in understanding cell type specificity, spatiotemporal dynamics, and achieving precise clinical application. Future interdisciplinary research will further advance the role of immune-neural interaction as a key target for preventing and treating neurological diseases, providing more precise diagnostic and therapeutic strategies.

## 1 Introduction

Neuroinflammation stands as a pivotal pathological hub in nervous system diseases (1), encompassing neurodegenerative disorders and psychiatric conditions (2). Central to neuroinflammation is the bidirectional interaction between immune cells and neural cells (3, 4). This interaction modulates disease progression via mechanisms including cytokine signaling, synaptic regulation, and metabolic reprogramming (5, 6). While existing research has established that dysregulated immune-neural interaction amplifies pathological protein toxicity and impairs neural plasticity, the systemic integration of these interactions across different disease contexts and their translation to clinical practice remains incomplete (7).

Despite significant advances in understanding individual components of immune-neural crosstalk, critical knowledge gaps persist. First, the cell type specificity of interactions remains unclear—for instance, how distinct subtypes of disease-associated microglia (DAM) interact with specific neuron populations to drive disease-specific pathology (8). Second, the molecular details of peripheral-central immune communication are not fully elucidated, limiting the identification of peripheral targets (9). Third, clinical translation is hindered by insufficient specificity of biomarkers and the lack of personalized therapeutic strategies, as current interventions often target broad inflammatory pathways rather than disease-specific immune-neural dysregulation (10).

This review aims to address these gaps by centering on the core theme of “immune cell–neural cell interaction, neuroinflammation, nervous system diseases, and clinical translation.” It first dissects the molecular mechanisms of immune-neural interaction under physiological homeostasis and pathological disruption. Then, it uses representative diseases as case studies to reveal disease-specific mechanisms of interaction dysregulation. Finally, it summarizes the clinical translation progress of diagnostic biomarkers, therapeutic targets, and intervention strategies based on this interaction, and outlines future research directions. The overarching goal is to provide a systematic framework for understanding the role of immune-neural interaction in neuroinflammation-driven diseases and to guide interdisciplinary research at the intersection of neuroscience, immunology, and clinical medicine.

## 2 Neuroinflammation: from cellular crosstalk to disease pathology

### 2.1 Neuroinflammation: the core regulatory hub of nervous system diseases

Neuroinflammation is not an independent disease but an immune response process (11). It is characterized by the activation of central and peripheral immune cells, the release of proinflammatory factors, and the imbalance of the neural microenvironment (12). Neuroinflammation is usually induced by central nervous system (CNS) injury, infection, toxin stimulation, or autoimmune reactions (13). Initially, it acts as a protective response of the body to injury and threats. However, once the response becomes excessive or develops into chronic inflammation, it turns into a harmful process. This harmful process causes neuronal damage and functional abnormalities, thereby playing a key role in the occurrence and development of various nervous system diseases, including neurodegenerative diseases, brain injury, infectious diseases, autoimmune diseases, and psychiatric disorders (14).

In different diseases, neuroinflammation acts on different cellular targets and neural circuits, leading to significant differences in their clinical manifestations and pathological features. Neurodegenerative diseases exhibit diverse pathological and clinical characteristics, including selective vulnerability of specific brain regions and aggregation of different proteins (15). The core role of neuroinflammation in neurodegenerative diseases is to amplify the toxicity of pathological protein deposits and accelerate neuronal death. In contrast, depression and schizophrenia do not involve large-scale neuronal death or protein deposition. The core role of neuroinflammation in these diseases is to disrupt neuronal communication, plasticity, and function. Clinically, they share the features of impaired cognitive function, abnormal emotional/affective regulation, and decreased social function.

**Figure 1:**
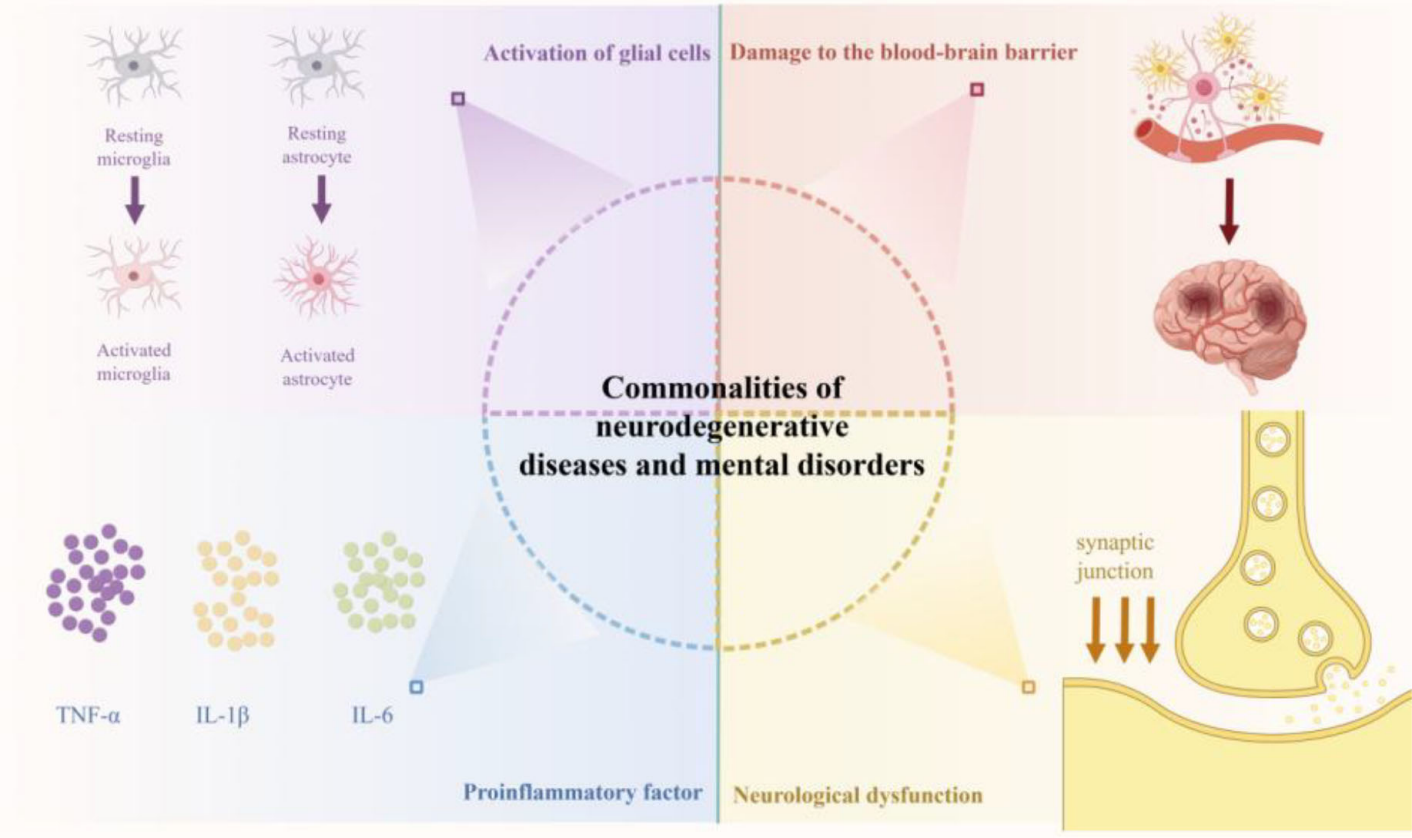
Common features of neurodegenerative diseases and psychiatric disorders. Schematic summarizing the common pathological features of neurodegenerative diseases (AD, PD) and psychiatric disorders (depression, schizophrenia). The core commonality is neuroinflammation, characterized by: 1) activation of microglia and astrocytes; 2) release of proinflammatory factors (TNF-α, IL-1β, IL-6); 3) BBB damage and peripheral immune cell infiltration; 4) synaptic dysfunction and neurological impairment. These features collectively drive disease progression, despite differences in specific pathological substrates.

Although neurodegenerative diseases and psychiatric disorders differ in clinical manifestations and pathological features, they both take neuroinflammation as the core regulatory hub of their pathological basis. The common features include: First, persistent activation of microglia and astrocytes, with the degree of activation positively correlated with disease severity (16); Second, increased levels of proinflammatory factors; Third, blood-brain barrier (BBB) damage, which opens the way for peripheral immune cells and inflammatory factors to infiltrate the CNS, forming a “peripheral-central loop” that synergistically amplifies neuroinflammation (17); and fourth, inflammation disrupting the function of different neural circuits by affecting synaptic function, leading to neural dysfunction. These common features are summarized in **Figure 1**.

### 2.2 Immune cell-neural cell interaction: the core effector unit of neuroinflammation

#### 2.2.1 Bidirectional communication between central immune cells and neurons

Neuroimmune communication is controlled by a complex, multi-layered network of cellular interactions and molecular signaling pathways. These interactions and pathways regulate the homeostasis and immune response of the CNS. The central mediators of this crosstalk include microglia, astrocytes, peripheral immune cells, and neurons. Together, they shape inflammatory and neuroprotective responses (18, 19).

On the core stage of neuroinflammation, central immune cells and neurons form a homeostatic unit that continuously conducts “internal communication.” Normal neural activity helps maintain the resting surveillance state of microglia (20). Astrocytes maintain the integrity of the BBB. Through the astrocyte-neuron lactate shuttle (ANLS), astrocytes transport lactate to neurons as an efficient energy substrate, providing immediate energy for synaptic transmission, maintenance of action potentials, and regeneration of glutamate in neurons (21).

**Figure 2:**
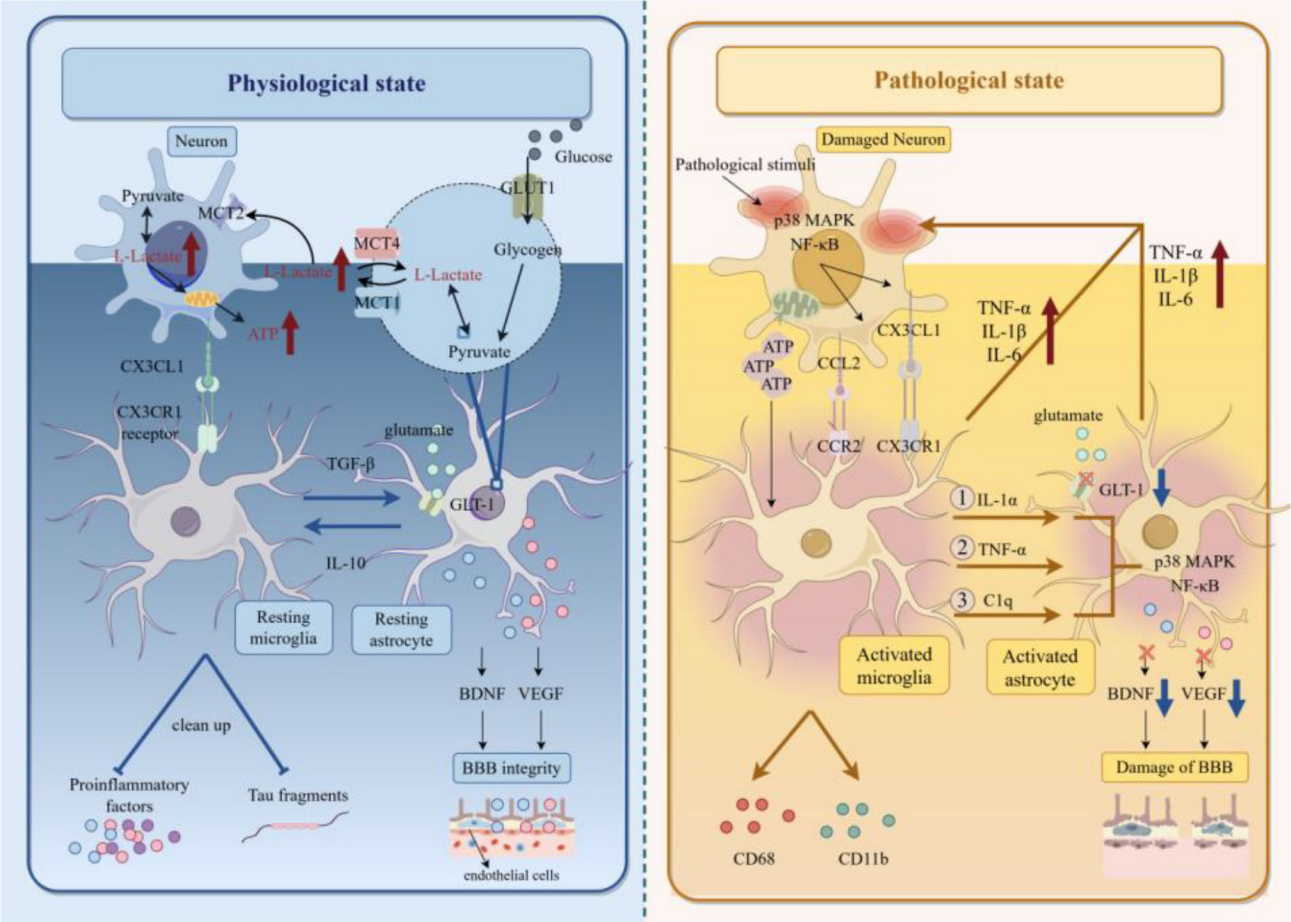
Bidirectional communication between microglia, astrocytes, and neurons under physiological and pathological conditions. Schematic comparing the bidirectional communication of microglia-astrocytes-neurons in physiological and pathological states. Physiological state: Neurons secrete CX3CL1 to maintain microglia in a resting surveillance state [synaptic pruning via Brain-derived neurotrophic factor (BDNF)]. Astrocytes provide metabolic support (including the conversion of glucose to lactate via MCT1/4 for neurons, and glutamate reuptake via GLT-1) and maintain BBB integrity. Pathological state: Damaged neurons release ATP and glutamate, activating microglia (via CCR2/CX3CR1) and astrocytes (via p38 MAPK/NF-κB). Activated glia secrete proinflammatory factors (TNF-α, IL-1β, IL-6) to impair neurons, reduce BDNF/Vascular endothelial growth factor (VEGF), and destroy BBB integrity (via CD68/CD11b upregulation).

The bidirectional communication between microglia, astrocytes, and neurons under physiological and pathological conditions is illustrated in **Figure 2**.

#### 2.2.2 Pathological activation of glial cells

Under pathological conditions, neurons express and release chemokines such as CCL2 and CX3C chemokine ligand 1 (CX3CL1). These chemokines directly bind to receptors on microglia, regulating the aggregation and phenotype of microglia (22, 23).

After activation, microglia release key cytokines such as IL-1α, Tumor necrosis factor-alpha (TNF-α), and C1q, which induce the formation of A1-type neuroinflammatory astrocytes (16). At the same time, activated microglia and astrocytes can release potent proinflammatory cytokines such as TNF-α, IL-1β, and IL-6. These cytokines act directly on neurons, impairing synaptic function, inhibiting long-term potentiation, inducing excitotoxicity, and even leading to cell apoptosis (24, 25). In Alzheimer’s disease (AD), they also promote the production of Amyloid-β (Aβ) and the hyperphosphorylation of tau protein (26).

#### 2.2.3 Interaction between peripheral immune cells and neural cells after central infiltration

**Figure 3:**
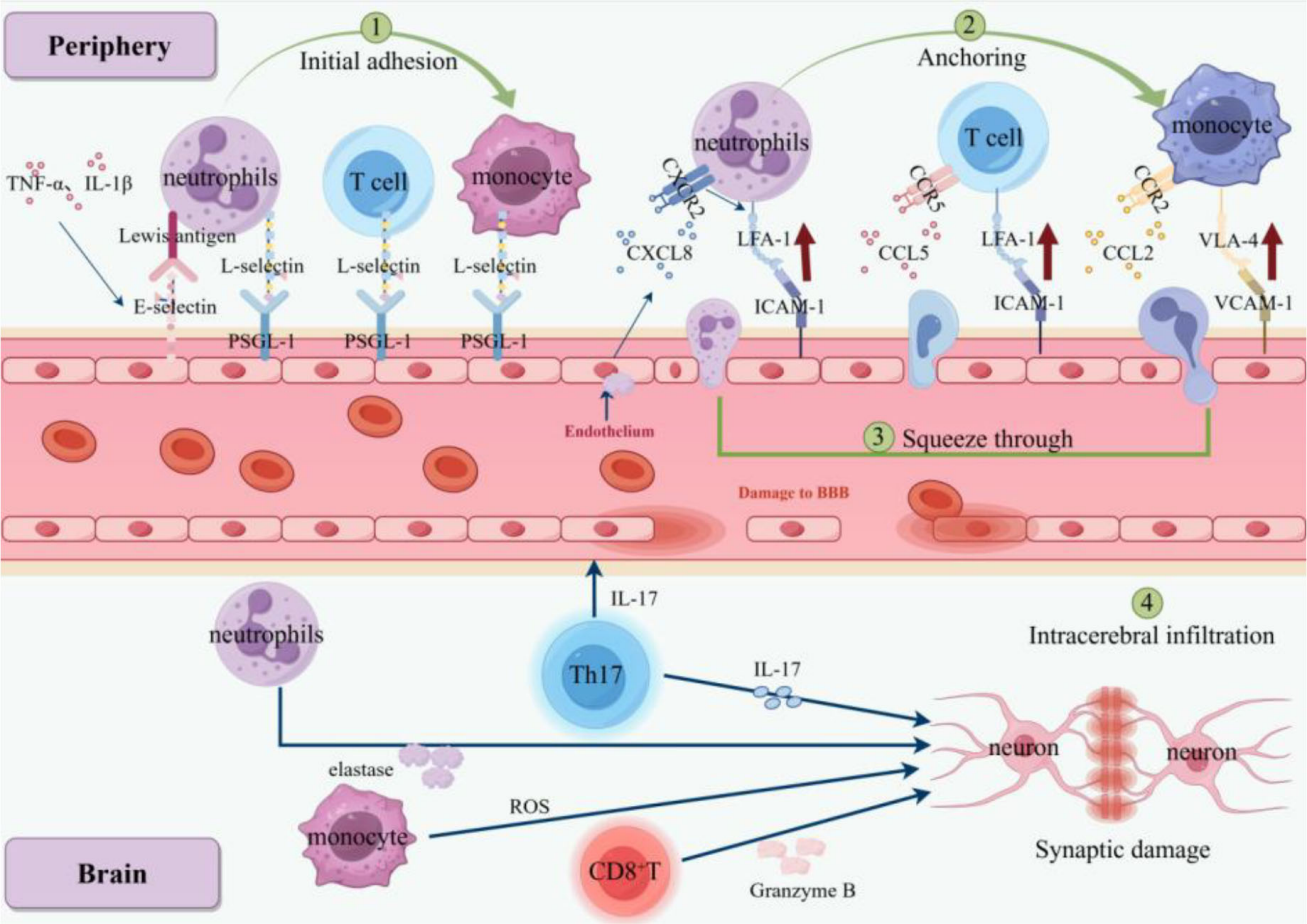
Pathological association between peripheral immune cell infiltration into the CNS (mediated by BBB damage) and neurons in AD. Schematic showing the process of peripheral immune cell infiltration into the CNS (mediated by BBB damage) and its interaction with neurons in AD. Peripheral proinflammatory factors (TNF-α, IL-1β) upregulate adhesion molecules (E-selectin, ICAM-1, VCAM-1) on endothelial cells. Neutrophils, T cells, and monocytes adhere to endothelial cells via L-selectin/P-selectin glycoprotein ligand-1 (PSGL-1), squeeze through the BBB, and infiltrate the brain. Neutrophils release elastase/ROS to damage neurons; CD8^+^ T cells secrete granzyme B to induce synaptic damage; Th17 cells produce IL-17 to further disrupt BBB integrity and neuronal function. This infiltration amplifies central neuroinflammation in AD.

Proinflammatory factors such as TNF-α, IL-1β, and IL-6 can cross the BBB through active transport mechanisms involving endothelial cell receptors. This further promotes the production of inflammatory mediators (27). When the BBB is damaged, peripheral immune cells infiltrate the central nervous system as “external variables”. Together with the internal “homeostatic unit”, they determine the final outcome of neuroinflammation. For example, in AD, brain microvascular endothelial cells highly express adhesion molecules such as Vascular cell adhesion molecule-1 (VCAM-1) and Intercellular adhesion molecule-1 (ICAM-1), which increases the permeability of the BBB (28). This increased permeability promotes the interaction between the peripheral immune system and the central nervous system, allowing peripheral immune cells to infiltrate the brain tissue. Infiltrated CD8^+^ T cells in the brain may directly cause neuronal dysfunction by releasing “granzyme B” (29). The mechanism involves regulating the expression of genes related to synaptic plasticity, thereby affecting the normal communication of neurons (30). IL-17 released by Th17 cells not only directly damages neurons but also impairs the integrity of the BBB (31). Around cerebral blood vessels, activated macrophages release reactive oxygen species (ROS), leading to oxidative stress in cerebral blood vessels and disrupting vascular function (32). This highlights the key role of perivascular monocytes/macrophages in cerebral amyloid angiopathy-mediated vascular permeability and microhemorrhage related to amyloid immunotherapy. They can amplify the local inflammatory environment and reshape the extracellular environment around vascular amyloid deposits (33). The pathological association between peripheral immune cell infiltration into the central nervous system (mediated by BBB damage) and neurons in AD is shown in **Figure 3**.

### 2.3 Molecular mediators of interaction: from cell-cell contact to soluble signals

**Figure 4:**
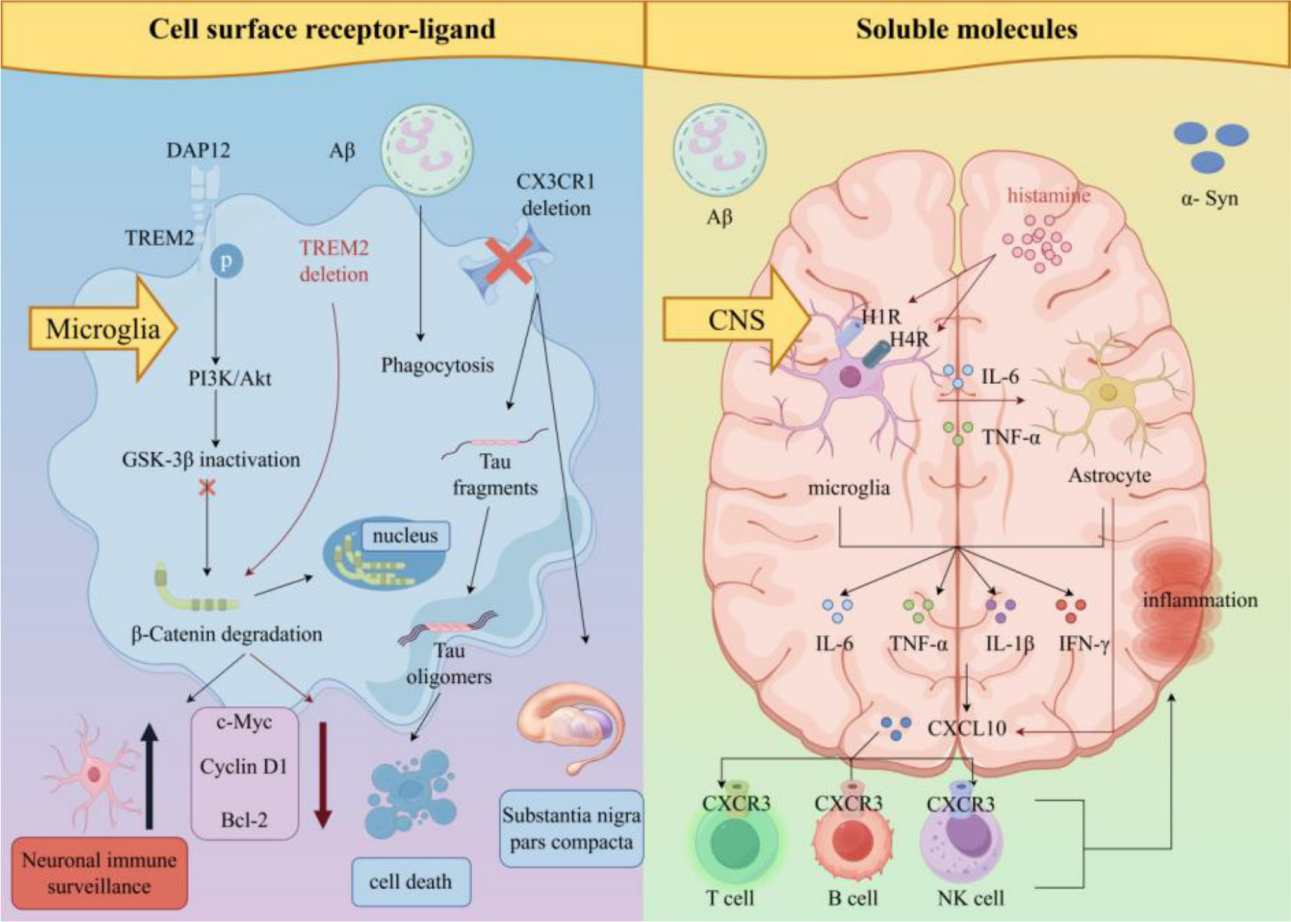
Cross-regulatory effects from “cell-cell contact” to “soluble signals”. Schematic illustrating the cross-regulatory network of immune-neural interaction via “cell-cell contact” and “soluble signals”. Cell-cell contact: Microglial TREM2 binds to DAP12, activating the PI3K/Akt pathway to inhibit GSK-3β, stabilize β-catenin, and regulate target genes (Cyclin D1, c-Myc, Bcl-2) for microglial survival and phagocytosis. CX3CR1 (microglia) interacts with neuronal CX3CL1 to modulate microglial activation. Soluble signals: Histamine (via H4R) promotes microglia to secrete IL-6/TNF-α, which induce astrocytes to produce CXCL10. CXCL10 binds to CXCR3 on T cells/B cells/NK cells to recruit them to the CNS. Proinflammatory factors (IL-6, TNF-α, IFN-γ) and pathological proteins (Aβ, tau, αSyn) further amplify inflammation and neuronal damage.

The interaction between immune cells and neural cells relies on two core types of molecular mediators. The cross-regulatory effects of immune-neural interaction from “cell-cell contact” to “soluble signals” are depicted in **Figure 4**.

#### 2.3.1 Cell surface receptor-ligand systems

In the brain, Triggering receptor expressed on myeloid cells 2 (TREM2) is one of the most highly expressed receptors in microglia. It regulates the phagocytic clearance of apoptotic neurons by microglia and the inflammatory response (34, 35). Under normal conditions, TREM2 binds to the transmembrane protein DNAX-activating protein of 12 kDa (DAP12), activating the downstream phosphoinositide 3-kinase/protein kinase B (PI3K/Akt) signaling pathway. In AD-related mutations, when TREM2 is deleted or dysfunctional, microglia exhibit G_1_/S phase arrest and increased apoptosis, and the inflammatory response becomes uncontrolled, ultimately exacerbating neuronal damage and neurodegenerative lesions (36, 37).

CX3C chemokine receptor 1 (CX3CR1) is a specific receptor for the chemokine CX3CL1, mainly expressed in microglia. Its signaling pathway shows significant functional differences in AD and Parkinson’s disease (PD) (38–40). In AD, inhibiting CX3CR1 signaling may accelerate the clearance of Aβ by activating microglia (41). However, if CX3CR1 signaling is interrupted, the pathological progression of tau protein is accelerated, increasing abnormal phosphorylation and aggregation of tau protein (42). In PD, the loss of the CX3CL1/CX3CR1 axis causes microglia to lose normal inhibitory regulation, leading to excessive activation that releases large amounts of proinflammatory factors, attacking dopaminergic neurons and exacerbating motor dysfunction (43).

#### 2.3.2 Soluble molecular network

In neuroinflammation, cytokines/chemokines and neurotransmitters/immune-transmitters together form a complex regulatory network. Under stimulation by neural injury or pathological proteins [Aβ, α-Synuclein (αSyn)], microglia and astrocytes first release proinflammatory factors such as TNF-α, IFN-γ, IL-1β, and IL-6 as the initiating signals of the inflammatory response (44–46). IFN-γ is a key factor inducing the expression of chemokines such as CXCL9, CXCL10, and CXCL11 (47–49).

The CXCL10-CXCR3 axis is one of the most critical chemotactic signaling pathways in central neuroinflammation. CXCL10 is secreted by astrocytes, microglia, and neurons. Through its receptor CXCR3, it attracts CD8+ T cells, B cells, NK cells, and other cells into the brain parenchyma, further amplifying the inflammatory response (50, 51).

Histamine is a typical “immune-neural” transmitter. It can act as a neuromodulator to affect neuronal activity and as an inflammatory mediator to regulate immune cells. Through H1R/H4R, histamine promotes microglia to produce proinflammatory factors, which can further induce astrocytes to produce CXCL10, forming a positive feedback loop that amplifies inflammation (52).

In summary, the cross-regulation of cytokines, chemokines, and neurotransmitters/immune-transmitters profoundly reveals the complex cross-talk between different systems in neuroinflammation.

### 2.4 Disease-specific pathological mechanisms of immune-neural dysregulation

**Figure 5:**
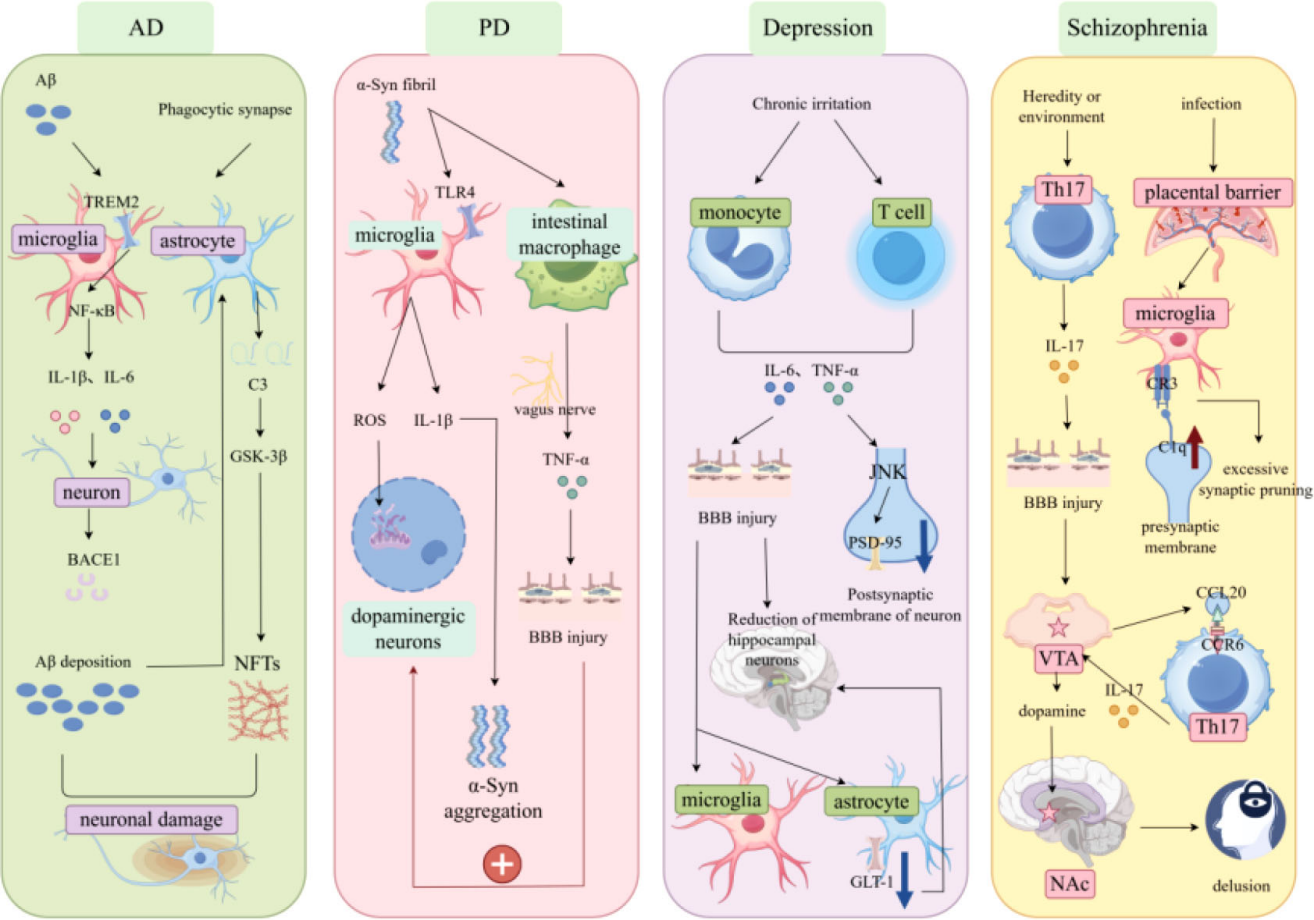
Mechanistic analysis of four major nervous system diseases driven by immune cell-neural cell interaction disorders. Schematic illustrating the core mechanisms of four major nervous system diseases (AD, PD, depression, schizophrenia) driven by immune cell-neural cell interaction disorders. AD: Aβ activates microglia via TREM2, triggering NF-κB-mediated release of IL-1β/IL-6; astrocytes secrete C3 to promote tau hyperphosphorylation (via GSK-3β) and Aβ deposition, leading to neuronal damage and NFT formation. PD: αSyn fibrils activate microglia via TLR4, releasing ROS/IL-1β; peripheral macrophages transmit inflammatory signals via the vagus nerve, exacerbating dopaminergic neuron loss. Depression: Chronic stress induces peripheral IL-6/TNF-α release; these cytokines activate microglia/astrocytes, reduce GLT-1 expression (glutamate excitotoxicity), and impair hippocampal neurogenesis. Schizophrenia: Maternal-fetal infection or gut dysbiosis activates microglia, causing excessive synaptic pruning (via C1q); Th17 cells infiltrate the CNS, release IL-17 to enhance VTA dopaminergic neuron excitability, and increase dopamine in the Nucleus accumbens, leading to delusions.

The mechanistic analysis of the four major nervous system diseases (AD, PD, depression, schizophrenia) driven by immune cell-neural cell interaction disorders is illustrated in **Figure 5**.

#### 2.4.1 Alzheimer’s disease: Immune-neural interaction and Aβ/tau pathology

**Figure 6:**
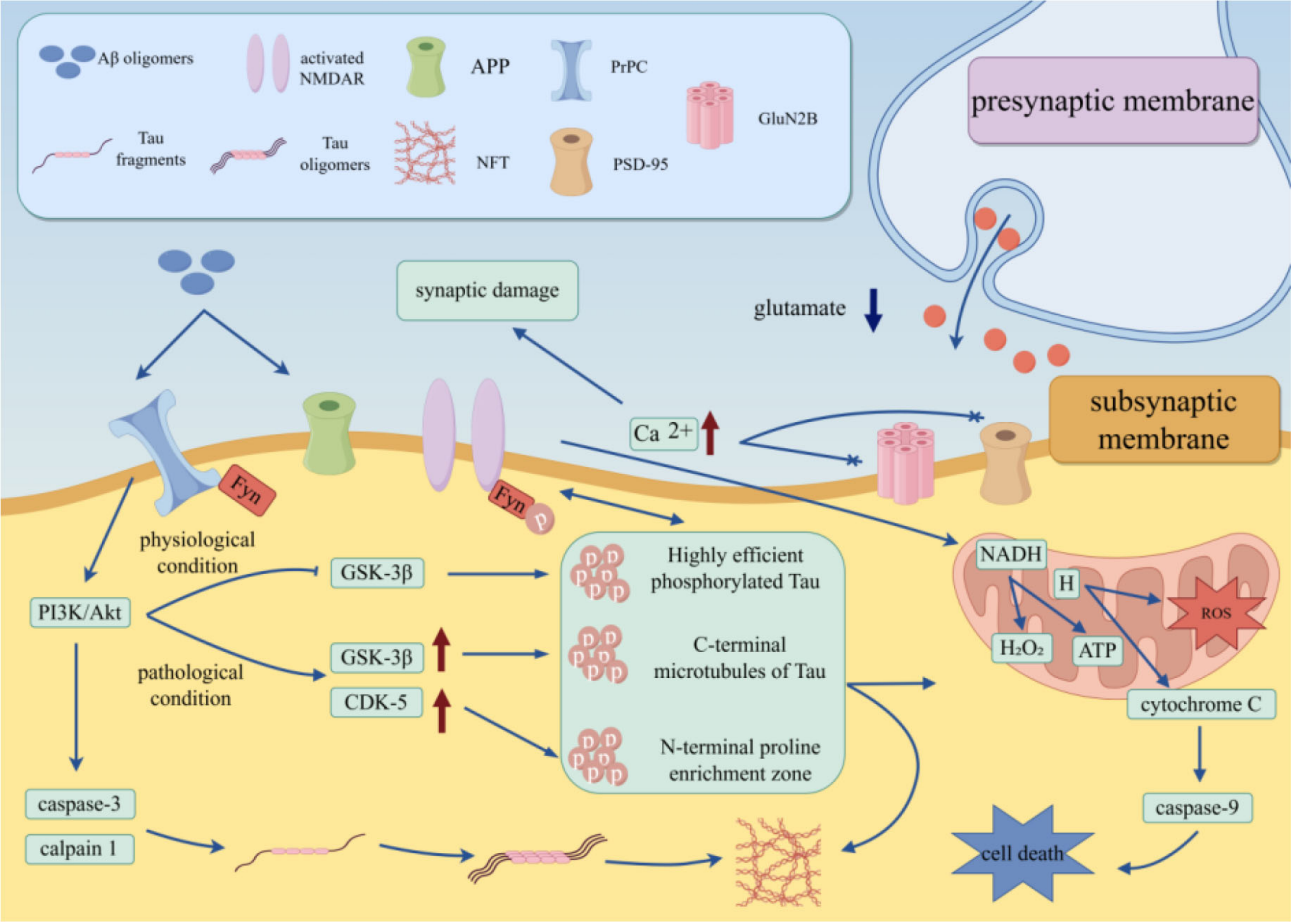
The synergistic effect of Aβ and tau protein in AD. Schematic showing the synergistic toxic mechanism of Aβ and tau protein in AD. Pathological Aβ oligomers bind to neuronal surface receptors to activate the PI3K/Akt pathway, which further triggers kinases (GSK-3β, CDK-5) to promote tau hyperphosphorylation. Hyperphosphorylated tau detaches from microtubules, aggregates into oligomers and NFTs, and stabilizes Aβ’s toxic conformation via “cross-seeding”. Additionally, Aβ-tau complexes activate the Fyn kinase-N-methyl-D-aspartate receptor (NMDAR) pathway, leading to abnormal Ca²^+^ influx, synaptic damage, mitochondrial dysfunction, and neuronal apoptosis (via caspase-3/9 activation).

The core pathology of AD is Aβ deposition and tau tangles. The synergistic effect of Aβ and tau protein in AD is illustrated in **Figure 6** (53, 54).

At the level of Aβ pathology, the imbalance between clearance and activation of Aβ by microglia is key: TREM2 mediates the phagocytosis of Aβ by microglia, but continuous phagocytosis activates the NF-κB pathway in microglia, leading to the release of IL-1β and IL-6 (55). These proinflammatory factors enhance the activity of neuronal β-secretase (BACE1) and promote Aβ production, while also disrupting the autophagic function of neurons, reducing Aβ clearance, and forming an “Aβ deposition-immune activation” vicious cycle (55).

**Figure 7:**
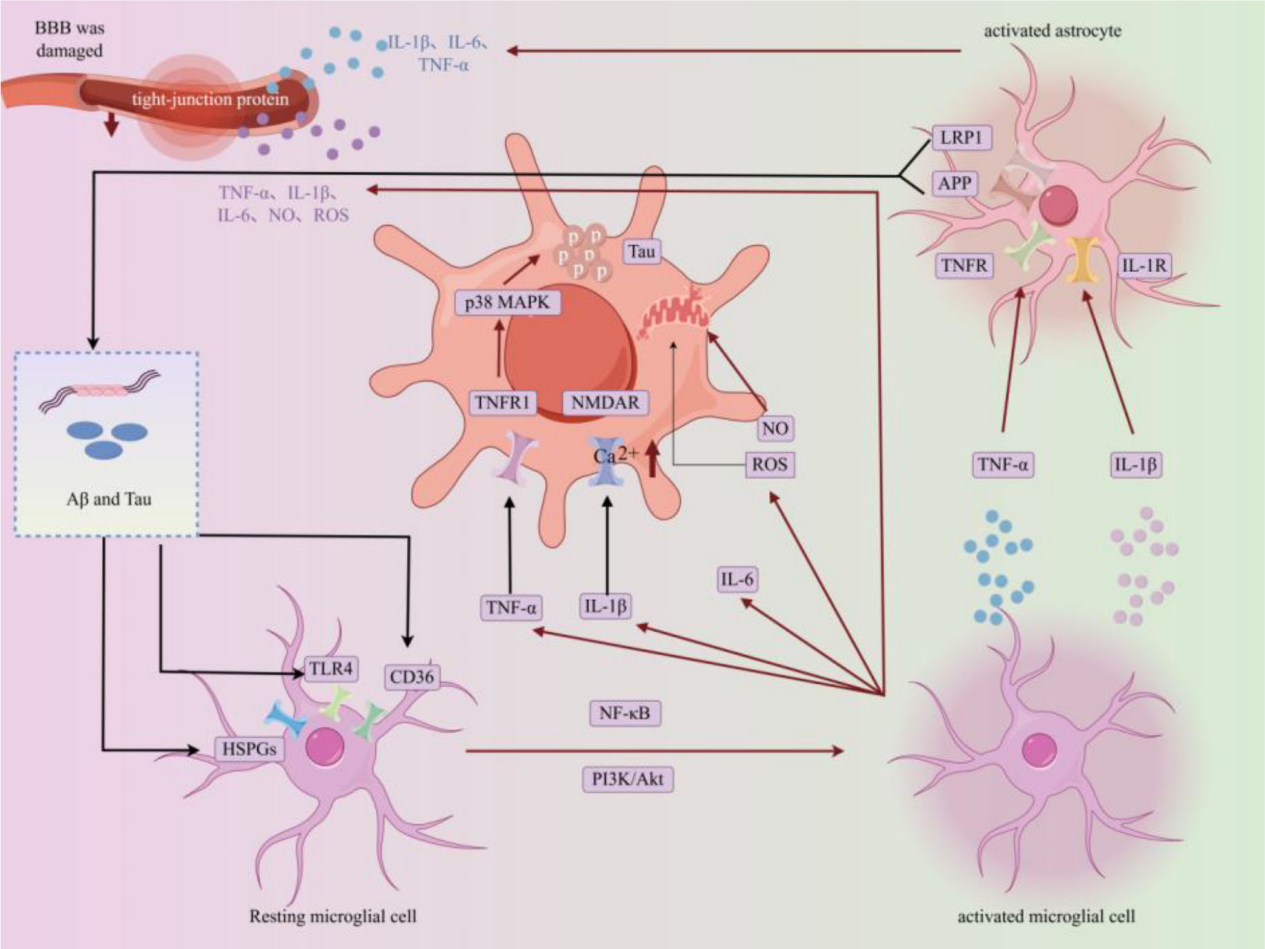
Glial inflammation jointly mediates BBB damage and pathological protein spread. Schematic illustrating how glial inflammation drives BBB damage and pathological protein (Aβ, tau) spread. Activated microglia (via TLR4/CD36) and astrocytes secrete proinflammatory factors (TNF-α, IL-1β, IL-6, NO, ROS), which downregulate tight-junction proteins in BBB endothelial cells to increase permeability. Meanwhile, these factors promote Aβ production (via BACE1 activation) and tau hyperphosphorylation (via p38 MAPK), and facilitate the spread of pathological proteins across brain regions. LRPI and TNFR/IL-1R on neurons mediate the toxic effects of glial-derived factors.

At the level of tau pathology, C3 complement secreted by A1-type polarized astrocytes not only phagocytoses synapses but also promotes the hyperphosphorylation of tau protein by activating the neuronal glycogen synthase kinase-3β (GSK-3β) pathway, accelerating the formation of neurofibrillary tangles (NFTs) (56, 57). The process by which glial inflammation mediates BBB damage and pathological protein spread is shown in **Figure 7**.

#### 2.4.2 Parkinson’s disease: Immune-neural interaction and dopaminergic neuron damage

**Figure 8:**
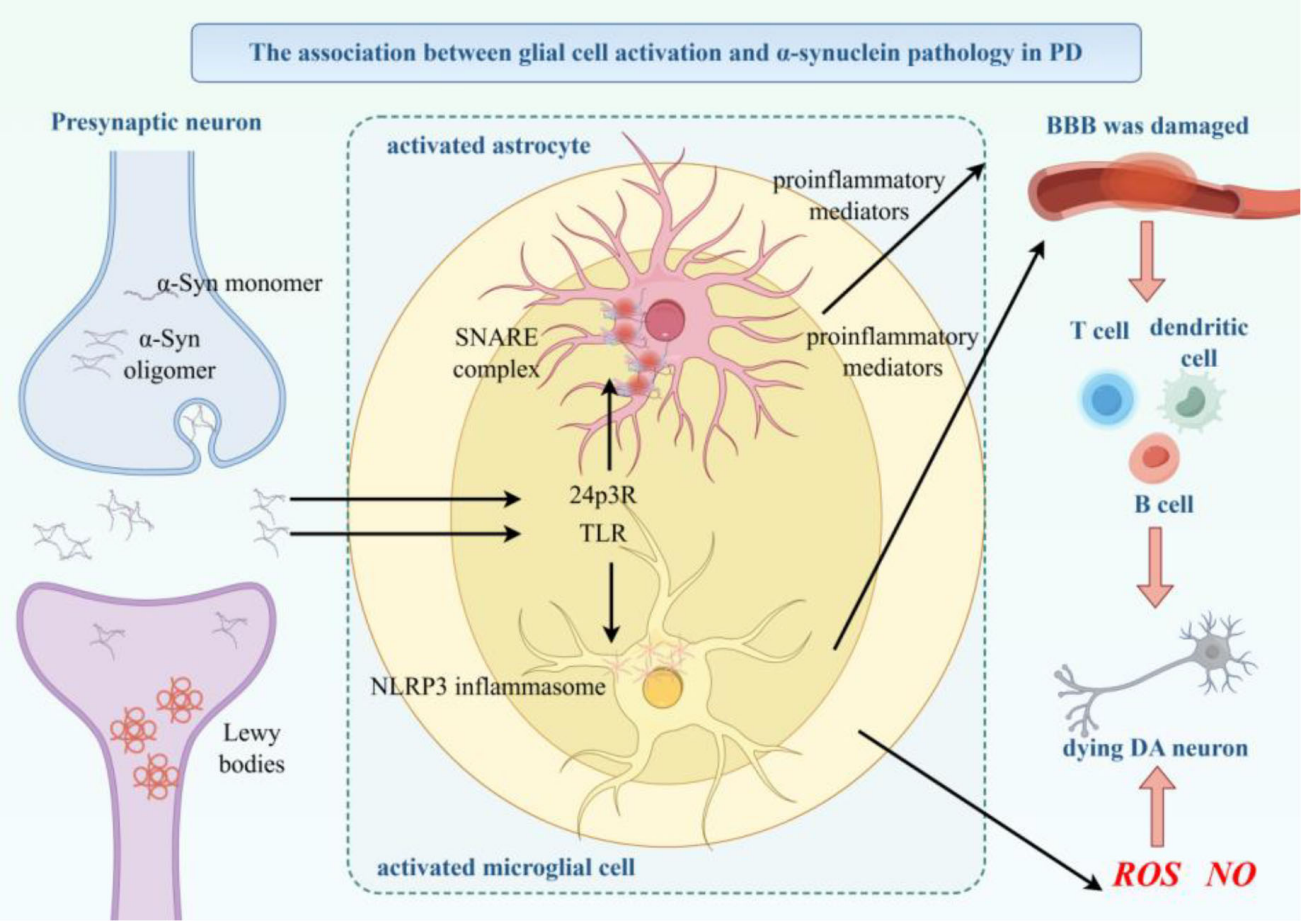
Pathological process of PD driven αSyn. Schematic depicting αSyn-mediated pathological progression in PD. Physiological αSyn monomers misfold into toxic oligomers and fibrils (Lewy bodies) under pathological conditions. These pathological αSyn forms activate microglia via TLRs and NLRP3 inflammasome, leading to ROS/NO production and dopaminergic neuron death. Activated astrocytes and infiltrated peripheral immune cells (T cells, dendritic cells, B cells via 24p3R) secrete proinflammatory mediators to exacerbate neuroinflammation. The loss of dopaminergic neurons in the substantia nigra pars compacta is the core pathological feature.

The core feature of PD is the selective death of dopaminergic neurons in the substantia nigra pars compacta (58). The pathological process of PD driven by αSyn is depicted in **Figure 8**.

At the central level, αSyn fibrils activate Toll-like receptors (TLRs) on microglia (59). The released reactive oxygen species (ROS) can directly oxidize the mitochondrial DNA (mtDNA) of dopaminergic neurons and inhibit the function of the respiratory chain (60). At the same time, IL-1β secreted by microglia enhances the misfolding and aggregation of αSyn in neurons (61).

**Figure 9:**
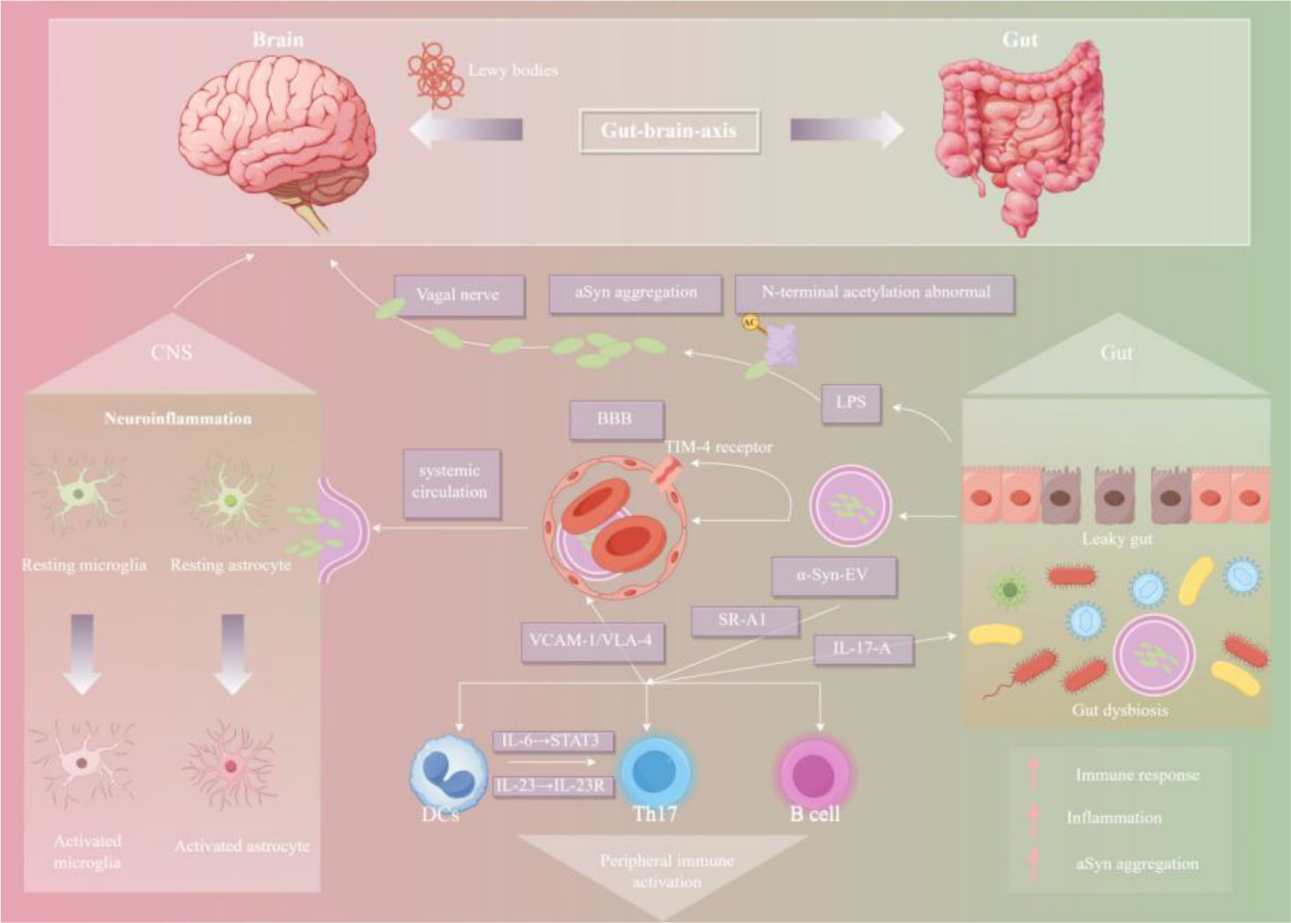
αSyn-mediated “gut-brain axis” vicious cycle. Schematic showing the αSyn-mediated “gut-brain axis” vicious cycle in PD. Misfolded αSyn accumulates in the gut, spreads retrogradely along the vagus nerve to the brainstem, and enters the systemic circulation via increased intestinal permeability (leaky gut). αSyn encapsulated in extracellular vesicles (EV-αSyn) penetrates the BBB or is transported by peripheral immune cells (DCs, Th17 cells) to the CNS, activating microglia and astrocytes. Gut dysbiosis triggers LPS release, which activates DCs to secrete IL-6/IL-23, driving Th17 cell differentiation and IL-17A production. This peripheral-central immune crosstalk amplifies neuroinflammation and αSyn aggregation.

At the peripheral level, misfolded αSyn accumulates in the intestines. It not only spreads retrogradely along the axons of the vagus nerve to the dorsal motor nucleus of the brainstem and then to the brain (62), but also increased intestinal permeability allows free αSyn or αSyn encapsulated in extracellular vesicles (EVs) to enter the systemic circulation (63–66). During systemic circulation, EV-αSyn can penetrate the BBB or be taken up by peripheral immune cells and then transported to the CNS. This αSyn-mediated neuro-immune interaction, occurring simultaneously in the central and peripheral systems, forms a “gut-brain axis” vicious cycle and jointly promotes the pathological progression of PD (67, 68). The αSyn-mediated “gut-brain axis” vicious cycle in PD is illustrated in **Figure 9**.

#### 2.4.3 Depression: Immune-neural interaction and abnormal neural plasticity

The core pathology of depression is abnormal neural plasticity. In peripheral-central signal transmission, chronic stress causes peripheral immune cells to release IL-6 and TNF-α. After these cytokines enter the CNS through the BBB, they activate microglia (69, 70). IL-6 released by proinflammatory microglia inhibits the proliferation of hippocampal neural stem cells and reduces the generation of new neurons. TNF-α promotes the degradation of synaptic-related proteins by activating the neuronal c-Jun N-terminal kinase (JNK) pathway, leading to synaptic loss (71, 72).

**Figure 10:**
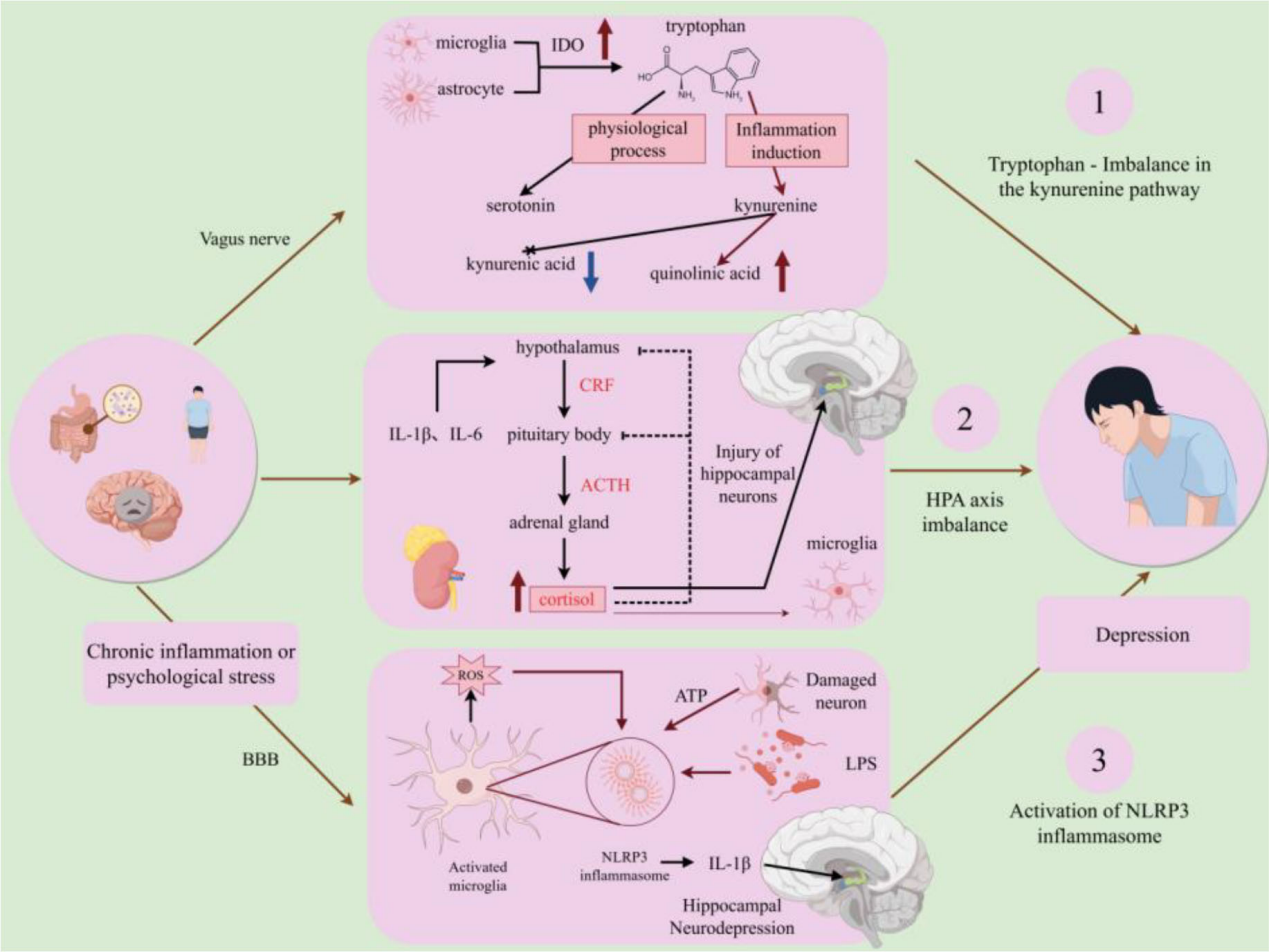
Pathological process of depression: From peripheral triggers to neuroinflammation mediated by the tryptophan-kynurenine pathway/HPA axis/NLRP3. Schematic illustrating the pathological mechanisms of depression. Peripheral triggers (chronic stress, LPS, BBB damage) activate microglia and astrocytes. Activated microglia shift tryptophan metabolism toward the kynurenine pathway, reducing serotonin synthesis and increasing neurotoxic quinolinic acid. Meanwhile, inflammatory signals activate the hypothalamic-pituitary-adrenal (HPA) axis, leading to excessive cortisol release and hippocampal neuron injury. The NLRP3 inflammasome in microglia is activated to secrete IL-1β, further impairing neural plasticity and neurogenesis, which manifest as depressive symptoms.

The pathological process of depression, from peripheral triggers to neuroinflammation mediated by the tryptophan-kynurenine pathway, Hypothalamic-pituitary-adrenal (HPA) axis, and NOD-like receptor family pyrin domain containing 3 (NLRP3) inflammasome (73–75), is shown in **Figure 10**.

At the same time, astrocyte dysfunction further exacerbates damage to neural plasticity. In depressed patients, the expression of glutamate transporter-1 (GLT-1) in astrocytes of the prefrontal cortex is decreased. Glutamate accumulation causes excitotoxicity, inhibiting the activity of the prefrontal cortex-hippocampal neural circuit, eventually manifesting as depressive symptoms such as low mood and anhedonia (76).

#### 2.4.4 Schizophrenia: immune-neural interaction and neural circuit disorder

The pathological core of schizophrenia is neural circuit imbalance. In the mechanism of positive symptoms (hallucinations, delusions), activated Th17 cells enter the CNS through the damaged BBB. Under chronic inflammatory conditions, the ventral tegmental area (VTA) of the midbrain secretes CCL20, and CCR6 on the surface of Th17 cells binds to CCL20, leading to directional migration of Th17 cells to the VTA region (77). Th17 cells accumulated in the VTA continuously release IL-17, which directly enhances the excitability of VTA dopaminergic neurons. Neurons with enhanced excitability release a large amount of dopamine into the nucleus accumbens, triggering positive symptoms (78).

In the mechanism of cognitive deficits, maternal-fetal infection during the embryonic period activates microglia in the fetal brain (79). Activated microglia overexpress C1q, leading to the marking of originally useful synapses. The complement receptor on the surface of microglia binds to C1q on synapses, initiating phagocytic signals and leading to excessive synaptic pruning (80, 81). This results in a decrease in the synaptic density of pyramidal neurons in the prefrontal cortex, affecting cognitive function. In addition, the secretion of neurotrophic factors such as glial cell line-derived neurotrophic factor (GDNF) by astrocytes is reduced, further inhibiting neuronal survival and synaptic plasticity (82).

**Figure 11:**
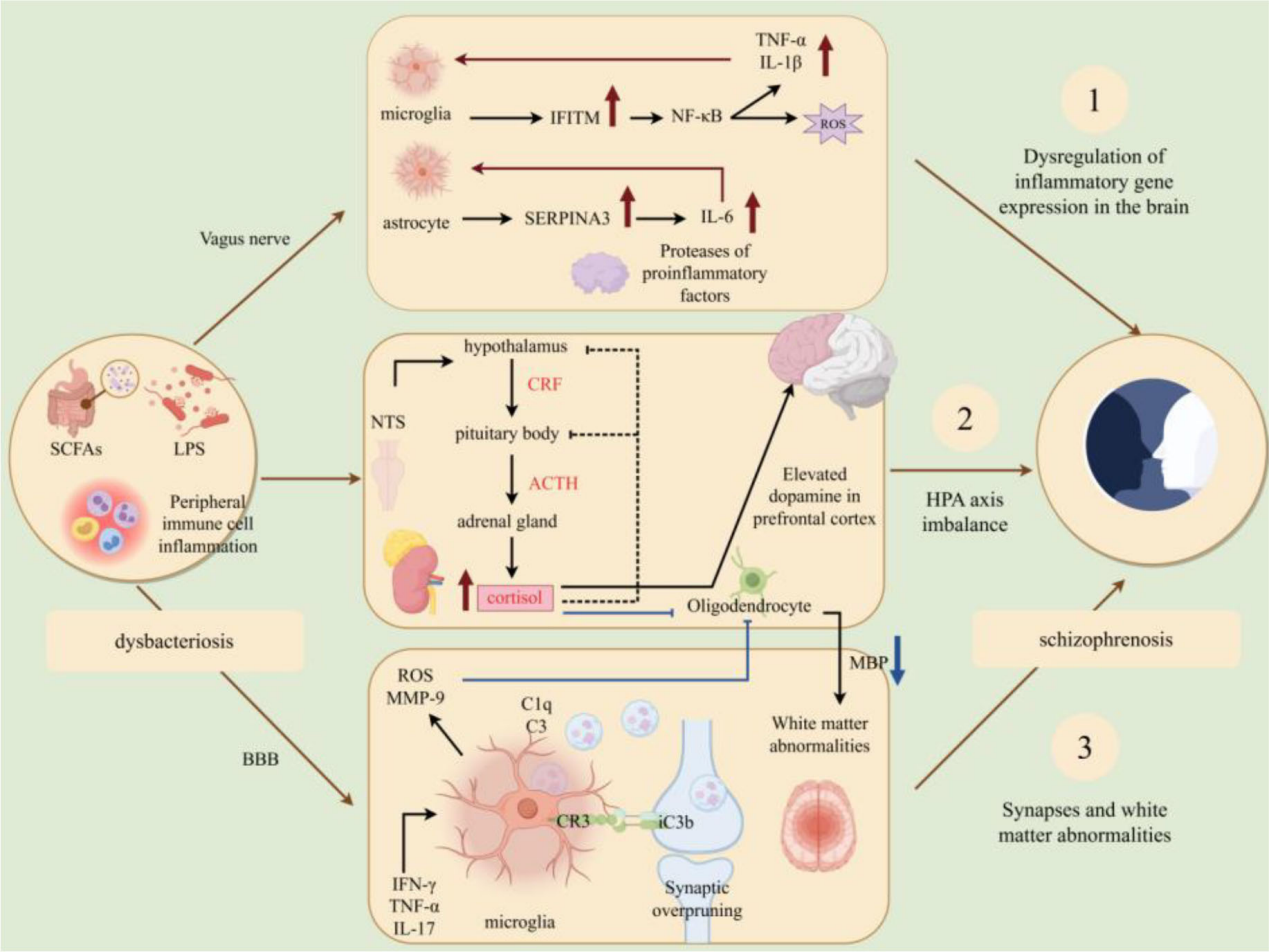
Pathological process of schizophrenia: From intestinal microbiota dysbiosis to neuroinjury mediated by gene expression dysregulation/HPA axis/synaptic and white matter abnormalities. Schematic depicting the pathological mechanisms of schizophrenia. Intestinal microbiota dysbiosis and LPS-induced intestinal inflammation transmit signals to the CNS via the vagus nerve, activating the nucleus tractus solitarius (NTS) and HPA axis. This leads to dopamine dysregulation in the prefrontal cortex, impaired myelin formation [reduced myelin basic protein (MBP)], and BBB abnormalities. Activated microglia overexpress C1q/C3 to induce excessive synaptic pruning, and upregulate inflammatory genes [Serpin family A member 3 (SERPINA3), Interferon-induced transmembrane protein (IFITM)]. Peripheral immune cell (T cell, macrophage) infiltration exacerbates neuroinflammation, resulting in neural circuit dysfunction and clinical symptoms.

The pathological process of schizophrenia, from intestinal microbiota dysbiosis to neuroinjury (83–85), is depicted in **Figure 11**.

## 3 Basic molecular mechanisms of immune cell-neural cell interaction

### 3.1 Interaction between microglia and neurons: “neuroimmune surveillance and homeostasis maintenance”

#### 3.1.1 Physiological state

In the “resting state”, microglia are not in a dormant or inactive state but actively and dynamically monitor the brain microenvironment. This resting phenotype is crucial for maintaining the homeostasis of the CNS. The “branched” resting phenotype of microglia maintains neuronal homeostasis through two pathways. On one hand, through their delicate protrusions, microglia contact synapses and participate in the precise “pruning” of redundant or weakened synapses. This process is also closely related to signaling pathways such as pro–brain-derived neurotrophic factor and its receptor p75 neurotrophin receptor (proBDNF-p75NTR) (86). On the other hand, Microglia secrete BDNF. They are an important source of BDNF in the brain. The BDNF they secrete is crucial for synaptic formation and plasticity during learning. It can promote neuronal survival and synaptic plasticity, and reduce oxidative stress damage by clearing neuronal metabolic waste (87).

#### 3.1.2 Pathological triggers

When neurons are damaged, they release DAMPs. These DAMPs activate microglia and induce their transformation into a proinflammatory phenotype (16, 88). Aβ aggregates induce the activation of microglia, leading to the release of nitric oxide, reactive oxygen species, cytokines and chemokines. All these substances may contribute to neuronal death (89). Aβ aggregates bind to TLRs and receptors for advanced glycation end products on microglia, inducing the transcriptional activation of downstream inflammatory response genes (90). On the other hand, TLR4 is a membrane-bound receptor mainly present on macrophages and microglia. The homodimerization of TLR-4 receptors leads to the activation of glycogen synthase kinase (GSK-3) and the transcription factor NF-κB. It can also induce the production of proinflammatory cytokines such as IL-1β, TNF-α, IL-6, and CXCL10 (91).

### 3.2 Interaction between astrocytes and neurons: “neurovascular unit and synaptic homeostasis”

#### 3.2.1 “Nutritional support” of astrocytes

Astrocytes act as the “metabolic support cells” of neurons. In glutamate metabolism, astrocytes actively clear approximately 90% of glutamate in the synaptic cleft through their highly expressed excitatory amino acid transporters (EAAT1/2). This not only terminates the excitatory signal of glutamate and prevents excitotoxicity but also, more importantly, the taken-up glutamate is converted into glutamine in astrocytes. Subsequently, glutamine is released and supplied to neurons as a direct precursor for the synthesis of glutamate and Gamma-aminobutyric acid (GABA) (92, 93).

In energy supply, astrocytes transport lactate produced by glucose metabolism to neurons through the “ANLS”. This lactate serves as an efficient energy substrate for neuronal synaptic transmission. Astrocytes take up glucose and undergo glycolysis to produce lactate. Then, through Monocarboxylate transporters (MCT1 and MCT4) on their cell membranes, they release lactate into the extracellular space. Neurons take up lactate through their specifically expressed MCT2 (94). Inside neurons, lactate is catalyzed by lactate dehydrogenase B into pyruvate, which enters the tricarboxylic acid cycle to produce a large amount of ATP. This meets the high energy demand of neurons during synaptic transmission and maintenance of neural excitability (95, 96).

#### 3.2.2 Pathological abnormalities

Under neuropathological conditions, activated microglia release specific inflammatory signals (TNF-α, IL-1α, C1q). These signals induce resting astrocytes to polarize into neurotoxic A1-type reactive astrocytes (97–99). Unlike normal astrocytes that play a supportive role, A1-type astrocytes release C3. C3 binds to the C3a receptor (C3aR) on neurons, leading to synaptic dysfunction and loss. This directly links the upregulation of C3 in astrocytes to synaptic damage. For example, in the context of AD, Aβ exposure activates the NF-κB pathway in astrocytes. One of the transcriptional targets of this pathway is C3 (100). Astrocytes release upregulated C3, which binds to the C3a receptor (C3aR) on neurons. The increased C3-C3aR signaling disrupts calcium homeostasis in neurons, ultimately damaging dendritic morphology, reducing synaptic density, and impairing excitatory synaptic transmission in the context of AD (100). This complement-dependent synaptic elimination driven by A1-type astrocytes is one of the key mechanisms of cognitive impairment in various neurodegenerative diseases and neuroinflammatory diseases.

### 3.3 Interaction between peripheral immune cells and central neural cells: “peripheral-central immune communication”

The damage to the integrity of the BBB is a prerequisite for peripheral T cells to enter the CNS. In the neuroinflammatory process of AD, the integrity of the BBB is damaged, and its permeability is increased. This creates conditions for peripheral immune cells to infiltrate the CNS. Among these cells, CD4+ T lymphocytes—including helper T cell subsets such as Th1 and Th17—can cross the damaged BBB and enter the brain parenchyma (101). These infiltrated T cells interact complexly with resident cells in the CNS. For example, Th17 cells secrete the key effector factor IL-17. This cytokine can activate microglia, induce their polarization into the proinflammatory phenotype, and promote the release of more inflammatory mediators. This significantly exacerbates the central inflammatory response and directly or indirectly causes neuronal damage and cognitive decline (94).

## 4 Clinical translation progress based on immune cell-neural cell interaction

### 4.1 Disease diagnosis: discovery and validation of biomarkers

#### 4.1.1 Central biomarkers

**Figure 12:**
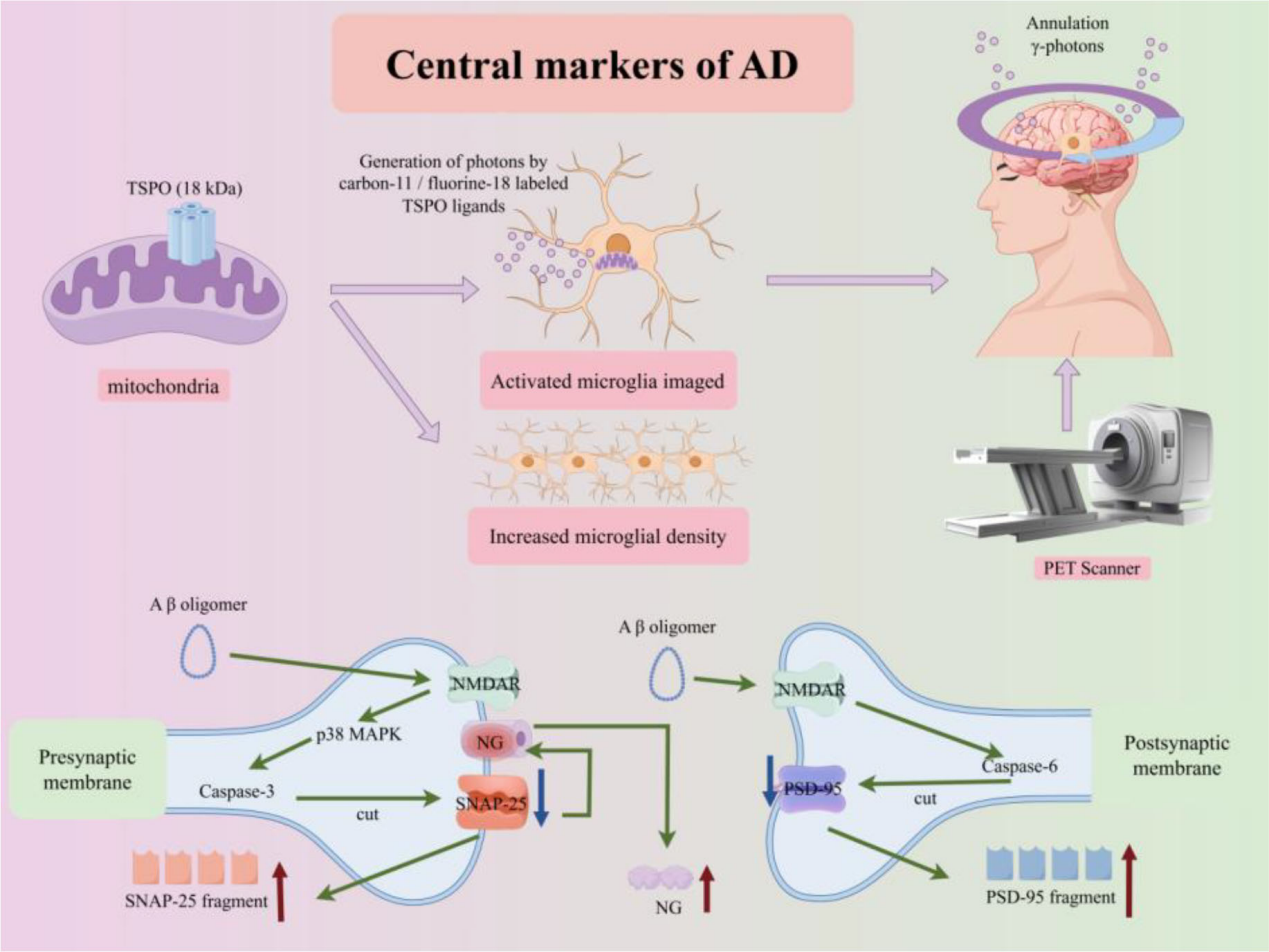
Central biomarkers of AD. Schematic depicting the central biomarkers of AD. Microglial activation biomarker (TSPO): TSPO in activated microglia is labeled with carbon-11/fluorine-18 radiotracers; PET imaging detects increased TSPO signals, indicating microglial activation and elevated density. Synaptic damage biomarkers: Pathological Aβ oligomers activate NMDAR, triggering caspase-3/6-mediated cleavage of presynaptic SNAP-25 and postsynaptic PSD-95/NG. CSF levels of SNAP-25 fragments, NG, and PSD-95 fragments reflect synaptic structural damage, correlating with cognitive decline.

In AD patients, the 18kD translocator protein (TSPO) can be imaged *in vivo* using positron emission tomography (PET). Increased TSPO PET signals indicate microglial activation and/or increased microglial density (102). Amyloid-induced regional TSPO expression occurs across all Braak stages and may be a driver of tau aggregation and spread in AD patients. TSPO in activated microglia is also closely related to neurodegeneration (103, 104).In addition, the level of the presynaptic membrane protein Synaptosomal-associated protein 25 (SNAP-25) in the cerebrospinal fluid (CSF) of AD patients is increased. It is positively correlated with the levels of postsynaptic membrane postsynaptic density protein 95 (PSD-95) and neurogranin (NG) (105). The higher the SNAP25 level, the lower the Mini-Mental State Examination score. This collectively reflects the overall structural damage of “presynaptic-postsynaptic” and can serve as a biomarker for presynaptic dysfunction (105). The central biomarkers of AD, including TSPO (for microglial activation) and synaptic damage-related proteins (SNAP-25, NG, PSD-95), are depicted in **Figure 12**.

#### 4.1.2 Peripheral biomarkers

Peripheral blood biomarkers are more clinically applicable due to their easy accessibility: IL-6 is a proinflammatory cytokine. The level of IL-6 in the peripheral blood of depressed patients is often increased, which may be involved in neuroinflammation and the development of depressive symptoms. Similar to IL-6, TNF-α is also often increased in depressed patients and is associated with inflammatory responses and abnormal neural function.

In addition, depression is associated with changes in several myeloid and lymphoid cells. These changes include increased average absolute counts of leukocytes, granulocytes, neutrophils, monocytes, CD4+ helper T cells, NK cells, CD19+ B cells, CD25+ and CD3+ HLADR+ activated T cells, but decreased relative percentages of lymphocytes, Th1 and Th2 cells. Therefore, identifying dysfunctional immune cell subsets can help in the design of future immunotherapy trials for depressed patients with immune dysfunction (106).

**Figure 13:**
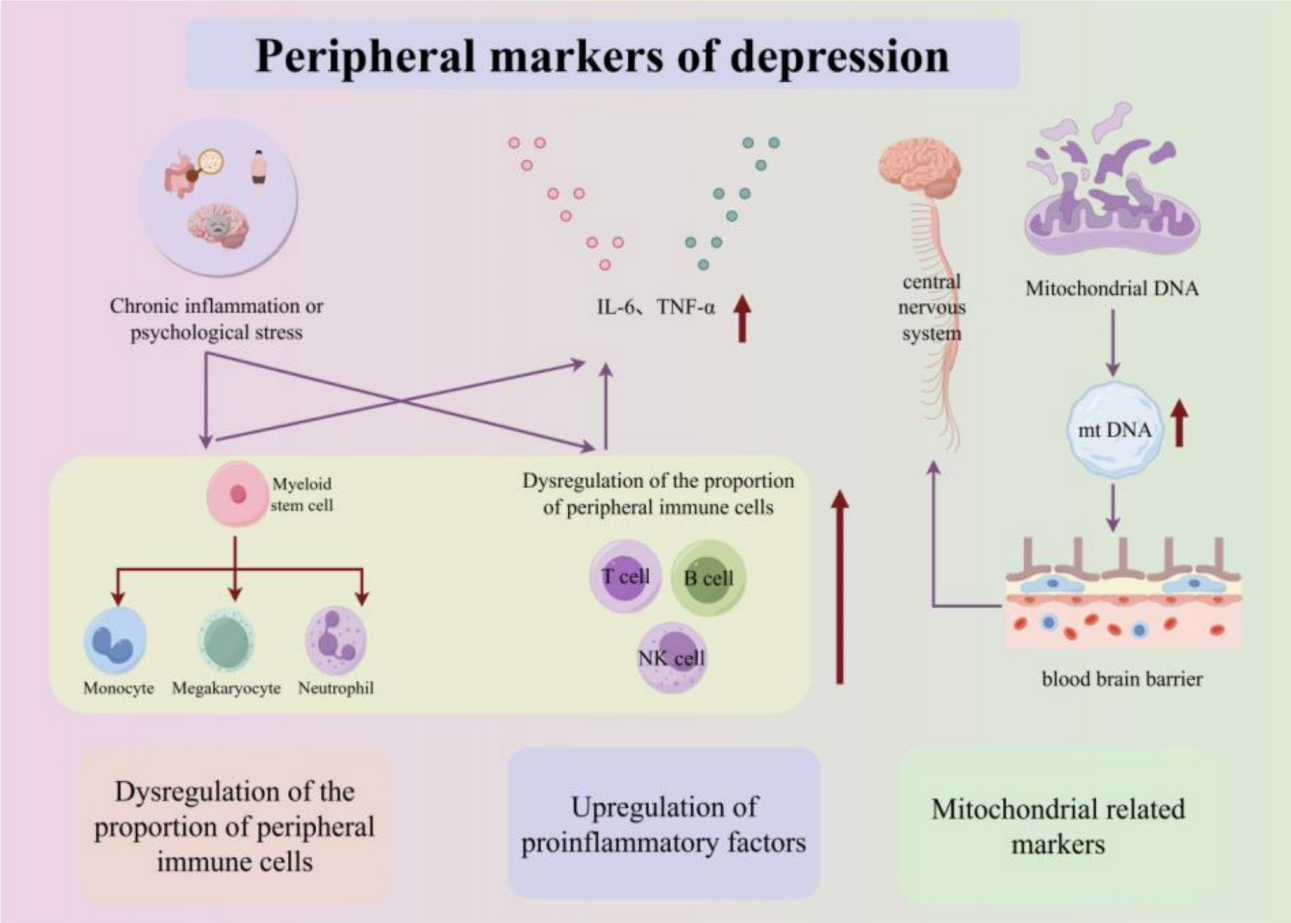
Peripheral markers of depression. Schematic summarizing the peripheral biomarkers of depression. Proinflammatory cytokines: Chronic stress or psychological stress induces peripheral immune cells to secrete IL-6 and TNF-α, which cross the BBB to activate central microglia/astrocytes. Immune cell subset dysregulation: Depression is associated with altered ratios of myeloid cells (increased monocytes, neutrophils) and lymphoid cells (increased NK cells, activated T cells; decreased Th1/Th2 cells). Mitochondrial markers: mtDNA is released into the peripheral circulation (stabilized by exosomes), serving as a neuroinflammatory biomarker for depression.

Mitochondria are considered the center of metabolism and biogenetics and interact with many pathological processes of major depressive disorder (107). MtDNA release serves as a key biological substrate and constitutes a pathway in neuroinflammatory diseases. After release, mtDNA can be carried in exosomes and transported to the extracellular space of the CNS and peripheral circulation. Detectable exosomes stabilize the enclosed mtDNA. Therefore, mtDNA in the peripheral circulation can be directly detected in clinical practice (108). The peripheral biomarkers of depression, including proinflammatory cytokines (IL-6, TNF-α), dysregulated immune cell subsets, and mtDNA, are shown in **Figure 13**.

### 4.2 Therapeutic targets: from “anti-inflammation” to “precise regulation of immune-neural interaction”

#### 4.2.1 Microglia targeting

Microglia are immune cells in the CNS. However, when stimulated by the external environment, microglia may transform into DAMs. Therefore, a series of new therapeutic strategies have been developed targeting DAMs.

**Figure 14:**
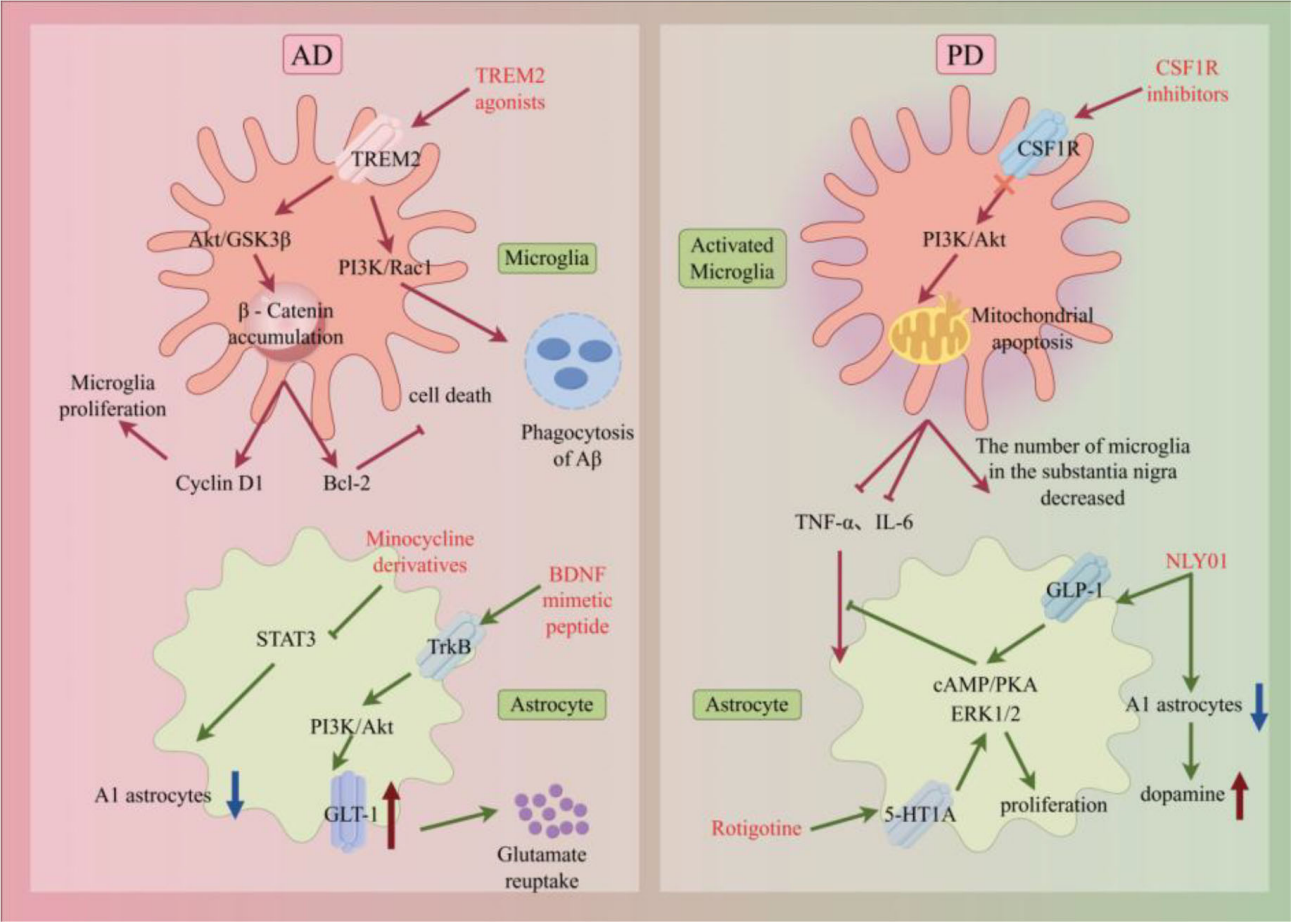
Targeted therapeutic strategies for microglia and astrocytes in neurological diseases. Schematic outlining targeted therapeutic strategies for microglia and astrocytes in neurological diseases (AD, PD). Microglia targeting: For AD, TREM2 agonists activate the PI3K/Akt pathway, stabilize β-catenin, and enhance microglial proliferation (Cyclin D1) and Aβ phagocytosis; for PD, CSF1R inhibitors reduce excessive microglial activation, decreasing TNF-α/IL-6 release and microglial apoptosis. Astrocyte targeting: Minocycline derivatives (STAT3 inhibitors) reduce A1-type astrocyte formation; GLT-1 agonists enhance glutamate reuptake; NLY01 blocks microglia-to-A1 astrocyte transformation; rotigotine (via 5-HT1A receptors) promotes astrocyte proliferation and BDNF secretion, protecting dopaminergic neurons.

TREM2 is a gene associated with AD risk. Its loss of function leads to microglial dysfunction and neurodegenerative diseases. TREM2 agonists can enhance the chemotaxis, phagocytosis, metabolism, and survival of microglia. This helps clear toxic Aβ oligomers in amyloid plaques, reduce neuronal synaptic damage, inhibit the abnormal aggregation of tau protein, and regulate the inflammatory response (109). Currently, oral small-molecule TREM2 agonists have entered phase II clinical trials for AD (110). In addition, colony-stimulating factor 1 receptor (CSF1R) is a key signaling pathway for microglial survival and proliferation. CSF1R inhibitors can reduce the excessive activation of microglia, thereby alleviating neuroinflammation. In PD, CSF1R inhibitors deplete microglia, thereby significantly reducing the formation of neurotoxic reactive astrocytes induced by α-Synuclein preformed fibril (αSyn PFF) (111). The targeted therapeutic strategies for microglia and astrocytes in neurological diseases are summarized in **Figure 14**.

#### 4.2.2 Astrocyte targeting

The core of astrocyte targets is to block A1-type polarization and synaptic toxicity: A1-type astrocyte inhibitors can reduce the production of A1-type astrocytes by inhibiting the Signal transducer and activator of transcription 3 (STAT3) pathway. Experiments in AD model mice show that these inhibitors can reduce C3 release in the hippocampus and improve synaptic density and cognitive function (112). Glutamate transporter GLT-1 agonists can enhance the glutamate reuptake capacity of astrocytes, alleviating glutamate excitotoxicity in the prefrontal cortex of depressed model rats. Currently, these agonists have entered phase I clinical trials (113). In addition, astrocyte metabolic support agents can enhance the function of ANLS, providing more energy for neurons and protecting dopaminergic neurons in PD models. NLY01 has potential clinical value in the treatment of neurodegenerative diseases involving A1 astrocyte activation. It can reduce the neurotoxic effect induced by αSyn PFF and reduce the loss of dopaminergic neurons and restore dopamine concentration in the striatum by blocking the transformation of microglia into A1 astrocytes (114). In addition to its dopaminergic effect, rotigotine can promote astrocyte proliferation through 5-HT1A receptors and enhance neuroprotection (115).

#### 4.2.3 Peripheral immune regulation

The damage to the BBB leads to the invasion of peripheral immune cells into the CNS, which is the core of neuroinflammation. Neuroimmune diseases have the characteristics of coexisting acute and chronic inflammation. Targeted inhibition of peripheral immunity can reduce lesion formation. Natalizumab—an effective drug for the treatment of multiple sclerosis—blocks integrins. It targets α4 integrins (mainly α4β1 and α4β7) on the surface of lymphocytes, blocking their binding to VCAM1 and thereby inhibiting the aggregation of lymphocytes to inflammatory sites (116). In addition, intestinal microbiota dysbiosis is closely related to impaired BBB integrity and enhanced neuroinflammatory responses. The loss of intestinal microbiota leads to abnormal stress responses, changes in neurotransmitter levels, and neurodevelopmental disorders. This further emphasizes the complex interaction between the microbiota, immunity, and the nervous system (117).

For example, in depression, immune signals derived from the intestine are also crucial. Transplanting fecal microbiota from patients with major depression into germ-free mice can induce depressive-like behaviors and is accompanied by increased levels of proinflammatory cytokines in the hippocampus. This provides ideas for the development of targeted drugs for “gut-brain axis” peripheral immune communication (118).

### 4.3 Clinical intervention strategies: exploration of emerging therapies

#### 4.3.1 Immunometabolic regulation

Immunometabolic regulation improves neural function by altering the metabolic phenotype of immune cells: Studies have shown that supplementation with itaconate derivatives (OI) can improve neural function. OI can restore the oxidative metabolism of glucose, glutamine, and fatty acids in microglia and enhance mitochondrial function. Therefore, OI treatment significantly inhibits the proinflammatory activation of microglia, reduces neuroapoptosis, and improves long-term neural function (119). Ketone body supplementation can alleviate the inflammatory response in AD by inhibiting the NLRP3 inflammasome. Higher circulating β-Hydroxybutyrate (BHB) levels in AD patients are associated with increased cognitive test scores. BHB has been shown to alter the disease process in AD mouse models through unknown mechanisms (120).

#### 4.3.2 Cell therapy

In CNS diseases, damage usually leads to the loss of neurons and glial cells and the destruction of neural circuits that are crucial for function (121). Stem cell therapy aims to address these issues by introducing cells that can differentiate into neurons and glial cells, thereby replacing lost or damaged tissue and promoting regenerative processes. This therapy has transformative potential for the treatment of neurodegenerative diseases through various therapeutic mechanisms (122). For PD, transplantation of iPSC-differentiated dopaminergic neurons can replenish lost neural cells and improve motor symptoms. Currently, multiple clinical trials have initially verified the safety and efficacy of stem cell therapy in the field of PD treatment (123).

#### 4.3.3 Neural regulation combined with immune intervention

**Figure 15:**
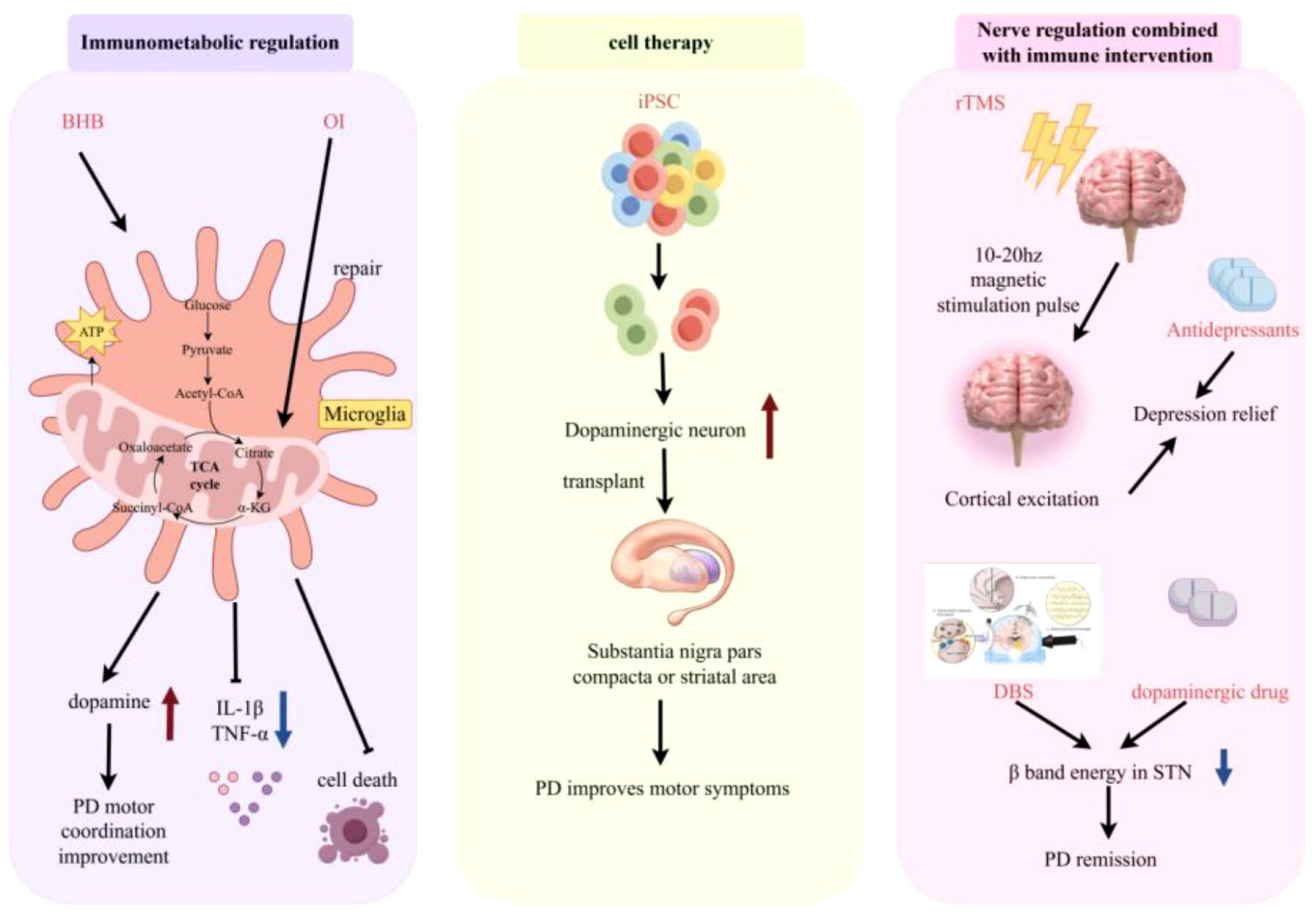
Mechanisms of neural regulation combined with immune intervention, immunometabolic regulation, and cell therapy. Schematic illustrating the mechanisms of three emerging clinical interventions for neurological diseases. Immunometabolic regulation: BHB and OI shift microglial metabolism from glycolysis to oxidative phosphorylation (TCA cycle), reducing proinflammatory activation (TNF-α/IL-1β) and improving PD motor coordination. Cell therapy: Transplantation of iPSC-derived dopaminergic neurons into the substantia nigra pars compacta or striatum replenishes lost cells, restoring dopamine levels and alleviating PD motor symptoms. Neural regulation combined with immune intervention: rTMS (10–20 Hz) enhances cortical excitability, and when combined with antidepressants, relieves depression; DBS plus dopaminergic drugs reduces β-band energy in the STN, ameliorating PD symptoms.

The combination of neural regulation technology and immune intervention can enhance the therapeutic effect: Repetitive transcranial magnetic stimulation (rTMS) can release a large number of repeated magnetic stimulation pulses and regulate the excitability of cerebral cortical neural tissue (124). Clinical studies have shown that combined treatment with “TMS + antidepressants” can increase the remission rate of depression, with a fast onset and safe efficacy (125). Abnormally increased beta-band (13–35 Hz) energy in the subthalamic nucleus (STN) is one of the pathophysiological characteristics of PD and is closely related to the severity of PD motor symptoms (126). Studies have shown that both deep brain stimulation (DBS) and dopaminergic drugs can effectively reduce beta-band energy in the STN, thereby alleviating PD symptoms. Combined treatment with drugs and DBS improves motor performance more significantly than drug treatment or DBS alone and produces more extensive beta-band inhibition (127). The mechanisms of emerging clinical interventions—including immunometabolic regulation, cell therapy, and neural regulation combined with immune intervention are illustrated in **Figure 15**.

## 5 Future challenges and research directions

### 5.1 Key scientific issues in basic research

The disorder of immune-neural interaction has “time dependence” and “spatial specificity”: In the early stage of AD, microglia mainly phagocytose Aβ. In the late stage, due to the continuous activation of microglia, the inflammasome is activated and proinflammatory components are released (128). In depressed patients, astrocyte dysfunction is the main feature in the prefrontal cortex, while microglial activation is the main feature in the hippocampus (129). In addition, in the parahippocampal gyrus of AD patients, upregulated proteins are mainly concentrated in immune response and glial cell activation pathways. This suggests that inflammation plays an important role in the pathological process of this brain region. The prefrontal cortex participates in the maintenance of cognitive function and disease progression through neurotransmitter balance and neural plasticity regulation (130). Therefore, in the future, it is necessary to establish a dynamic research model of “disease timeline-brain region spatial axis” to clarify the key time window and core brain region of interaction disorders, providing a basis for “early intervention” and “regional targeting”.

### 5.2 Bottlenecks and breakthrough points in clinical translation

#### 5.2.1 “Clinical specificity and sensitivity” of biomarkers

Existing biomarkers have the problem of “insufficient specificity”. For example, increased serum IL-6 is not only found in depression but also in infections and autoimmune diseases. Increased CSF TSPO is not only found in AD but also in brain trauma (131). However, increased TSPO is a non-specific manifestation of various nervous system diseases. AD or brain trauma cannot be diagnosed solely based on this indicator. Clinical diagnosis needs to be made comprehensively based on the patient’s medical history, symptoms, imaging examinations and other biomarkers. Therefore, in the future, it is necessary to develop “multimodal biomarkers”. Studies based on biobanks such as UKB, FinnGen, and SAIL have shown that compared with univariate analysis, integrating clinical data of digestive, endocrine, nutritional, and metabolic disorders, genetic risk scores, and proteomic data into prediction models (including multiple features) shows superior performance in predicting AD and PD (132).

### 5.2.2 “Personalization and timeliness” of intervention strategies

Traditional non-targeted intervention is prone to cause serious side effects. For example, the anti-total tau antibody 13G4 can reduce tau pathology in PS19 mice but causes obvious motor dysfunction. However, precision therapy based on patient immune phenotype can effectively avoid this problem. The team of Zhao Yingjun from Xiamen University developed a monoclonal antibody mAb2A7 that specifically targets p-tau217. By matching the tau pathology-specific immune phenotype of AD patients, it reduces neurodegeneration without damaging motor function (133). Similarly, the anti-CD20 monoclonal antibody ocrelizumab targets the immune phenotype characteristics of patients with multiple sclerosis and has become a first-line precision therapy drug in clinical practice. This confirms the feasibility and timeliness of formulating intervention plans based on immune phenotype (134).

### 5.3 Demands for technological and methodological innovation

#### 5.3.1 Single-cell multi-omics and spatial transcriptomics

Traditional bulk sequencing is difficult to resolve the heterogeneity of immune cell-neural cell interaction in nervous system diseases. There is an urgent need to map the “cell-molecular spatial map” through technology integration. A study on single-nucleus multi-omics of the prefrontal cortex in patients with advanced AD by the Vivek Swarup team from the University of California, Irvine showed that combining snATAC-seq and snRNA-seq technologies can identify disease-related transcription factors such as ETS1 and SPI1 in microglia, as well as the Glial fibrillary acidic protein (GFAP) state transition trajectory of astrocytes. This provides a basis for deciphering the molecular regulatory network of cell interaction (135). In addition, the development of data integration tools such as TSO-his has enabled the linked analysis of single-cell and spatial transcriptomic data. The experience of successfully reconstructing the colocalization relationship of cell types in thymus tissue provides a reference technical framework for mapping the spatial interaction pattern of immune-neural cells in the brain (136).

#### 5.3.2 Novel animal models and organoid models

Traditional mouse models are difficult to reproduce the characteristics of human central immune-neural interaction due to species differences. Humanized co-culture models have become a breakthrough direction. A study published in Mol Psychiatry by a team from Purdue University showed that the vascularized neuroimmune organoids they developed integrate neurons, microglia, and vascular endothelial cells. After treatment with brain tissue extracts from AD patients, pathological interaction processes such as phagocytosis of Aβ particles by microglia, upregulation of the proinflammatory factor IL-6, and synaptic damage can be observed. This provides a new pathophysiologically relevant model for AD research (137). Similarly, the 3D neurovascular unit bionic chip constructed by the team of the Dalian Institute of Chemical Physics, Chinese Academy of Sciences simulates the BBB microenvironment by co-culturing various human-derived cells. It successfully reproduces the inflammatory cascade reaction of microglial activation and peripheral immune cell infiltration after Herpes simplex virus type 1 (HSV-1) infection. This confirms the advantages of humanized models in deciphering immune-neural interaction mechanisms (138).

## 6 Conclusion

The bidirectional interaction between immune cells and neural cells is the core effector unit of neuroinflammation. Under physiological conditions, it maintains CNS homeostasis via microglial surveillance, astrocytic metabolic support, and BBB integrity. When dysregulated, it drives neuroinflammation through receptor-ligand systems and soluble signals.

This dysregulation manifests distinctly across diseases: AD features Aβ/tau synergy amplified by microglial TREM2 dysfunction and astrocytic C3; PD involves αSyn-driven microglial activation and gut-brain axis inflammation; depression involves peripheral cytokines triggering central neuroinflammation and impairing neurogenesis; schizophrenia features complement-mediated synaptic pruning and Th17 infiltration.

Clinically, targeting this interaction has yielded progress, including TREM2 agonists, CSF1R inhibitors, GLT-1 activators, immunometabolic regulation, and cell therapy. However, issues such as “cell subtype-specific mechanisms” and “clinical precision application” still need to be addressed. In the future, interdisciplinary research in neuroscience, immunology, and clinical medicine will promote “immune-neural interaction” to become a key target for the prevention and treatment of neurological diseases, providing more precise diagnosis and treatment strategies for patients.

## Glossary

AD: Alzheimer’s disease
ANLS: Astrocyte-neuron lactate shuttle
APOE: Apolipoprotein E
Aβ: Amyloid-β
BBB: Blood-brain barrier
BDNF: Brain-derived neurotrophic factor
BHB: β-Hydroxybutyrate
CNS: Central nervous system
CSF: Cerebrospinal fluid
CSF1R: Colony-stimulating factor 1 receptor
CX3CL1: CX3C chemokine ligand 1
CX3CR1: CX3C chemokine receptor 1
DAM: Disease-associated microglia
DAMPs: Damage-associated molecular pattern
DAP12: DNAX-activating protein of 12 kDa
DBS: Deep brain stimulation
EAAT: Excitatory amino acid transporter
EV: Extracellular vesicle
GABA: Gamma-aminobutyric acid
GDNF: Glial cell line-derived neurotrophic factor
GFAP: Glial fibrillary acidic protein
GLT-1: Glutamate transporter-1
GSK-3β: Glycogen synthase kinase-3β
HPA: Hypothalamic-pituitary-adrenal
HSV-1: Herpes simplex virus type 1
ICAM-1: Intercellular adhesion molecule-1
IFITM: Interferon-induced transmembrane protein
JNK: c-Jun N-terminal kinase
MBP: Myelin basic protein
MCT: Monocarboxylate transporter
mtDNA: Mitochondrial DNA
NFT: Neurofibrillary tangle
NG: Neurogranin
NLRP3: NOD-like receptor family pyrin domain containing 3
NMDAR: N-methyl-D-aspartate receptor
NTS: Nucleus tractus solitarius
OI: Itaconate derivative
PD: Parkinson’s disease
PET: Positron emission tomography
PI3K/Akt: Phosphoinositide 3-kinase/protein kinase B
PSD-95: Postsynaptic density protein 95
PSGL-1: P-selectin glycoprotein ligand-1
ROS: Reactive oxygen species
rTMS: Repetitive transcranial magnetic stimulation
SERPINA3: Serpin family A member 3
SNAP-25: Synaptosomal-associated protein 25
STAT: Signal transducer and activator of transcription
STN: Subthalamic nucleus
TLR: Toll-like receptor
TNF-α: Tumor necrosis factor-alpha
TREM2: Triggering receptor expressed on myeloid cells 2
TSPO: 18 kDa translocator protein
VCAM-1: Vascular cell adhesion molecule-1
VEGF: Vascular endothelial growth factor
VTA: Ventral tegmental area
αSyn: α-Synuclein
αSyn PFF: α-Synuclein preformed fibril.
